# A New Orchid Genus, *Danxiaorchis*, and Phylogenetic Analysis of the Tribe Calypsoeae

**DOI:** 10.1371/journal.pone.0060371

**Published:** 2013-04-04

**Authors:** Jun-Wen Zhai, Guo-Qiang Zhang, Li-Jun Chen, Xin-Ju Xiao, Ke-Wei Liu, Wen-Chieh Tsai, Yu-Yun Hsiao, Huai-Zhen Tian, Jia-Qiang Zhu, Mei-Na Wang, Fa-Guo Wang, Fu-Wu Xing, Zhong-Jian Liu

**Affiliations:** 1 South China Botanical Garden, Chinese Academy of Sciences, Guangzhou, China; 2 Shenzhen Key Laboratory for Orchid Conservation and Utilization, The National Orchid Conservation Center of China and The Orchid Conservation and Research Center of Shenzhen, Shenzhen, China; 3 Graduate University of Chinese Academy of Sciences, Beijing, China; 4 Institute of Tropical Plant Sciences and Orchid Research Center, National Cheng Kung University, Tainan City, Taiwan; 5 School of Life Science, East China Normal University, Shanghai, China; 6 Information Center of Renhua County, Shaoguan, China; 7 Center for Biotechnology and BioMedicine, Graduate School at Shenzhen, Tsinghua University, Shenzhen, China; 8 Landscape College of Fujian Agriculture and Forestry University, Fuzhou, China; 9 College of Forestry, South China Agricultural University, Guangzhou, China; Swiss Federal Institute of Technology (ETH Zurich), Switzerland

## Abstract

**Background:**

Orchids have numerous species, and their speciation rates are presumed to be exceptionally high, suggesting that orchids are continuously and actively evolving. The wide diversity of orchids has attracted the interest of evolutionary biologists. In this study, a new orchid was discovered on Danxia Mountain in Guangdong, China. However, the phylogenetic clarification of this new orchid requires further molecular, morphological, and phytogeographic analyses.

**Methodology/Principal Findings:**

A new orchid possesses a labellum with a large Y-shaped callus and two sacs at the base, and cylindrical, fleshy seeds, which make it distinct from all known orchid genera. Phylogenetic methods were applied to a matrix of morphological and molecular characters based on the fragments of the nuclear internal transcribed spacer, chloroplast *mat*K, and rbcL genes of Orchidaceae (74 genera) and Calypsoeae (13 genera). The strict consensus Bayesian inference phylogram strongly supports the division of the Calypsoeae alliance (not including *Dactylostalix* and *Ephippianthus*) into seven clades with 11 genera. The sequence data of each species and the morphological characters of each genus were combined into a single dataset. The inferred Bayesian phylogram supports the division of the 13 genera of Calypsoeae into four clades with 13 subclades (genera). Based on the results of our phylogenetic analyses, Calypsoeae, under which the new orchid is classified, represents an independent lineage in the Epidendroideae subfamily.

**Conclusions:**

Analyses of the combined datasets using Bayesian methods revealed strong evidence that Calypsoeae is a monophyletic tribe consisting of eight well-supported clades with 13 subclades (genera), which are all in agreement with the phytogeography of Calypsoeae. The Danxia orchid represents an independent lineage under the tribe Calypsoeae of the subfamily Epidendroideae. This lineage should be treated as a new genus, which we have named *Danxiaorchis*, that is parallel to *Yoania*. Both genera are placed under the subtribe Yoaniinae.

## Introduction

Orchidaceae is one of the largest families of angiosperms [1]. It has been said “The speciation rate in orchids is frequent because of their diverse flower and vegetable morphologies” [2]. Considerable attention has been given to their extraordinary pollination, multiple adaptive strategies to various habitats, and numerous dust-like seeds that lack endosperms [3], [4]. Previously, Orchidaceae has been divided into five subfamilies based on their morphological characters as follows: Apostasioideae, Cypripedioideae, Spiranthoideae, Orchidoideae, and Epidendroideae [5]. However, a phylogenetic analysis based on the internal transcribed spacer (ITS), *trn*L-F, and *mat*K sequences revealed that Spiranthoideae is a member of Orchidoideae and that *Vanilla* and its allies should be separated from Epidendroideae to form a new subfamily, Vanilloideae [6]–[8]. Epidendroideae is a highly evolved and diverse subfamily, in which a few species are mycotrophic and lack green leaves. Many holomycotrophic orchids are found in China, comprising approximately 20 genera. None of these orchids has a bisaccate labellum.

*Vanilla*, *Apostasia*, *Cyrtosia*, *Palmorchis*, *Selenipedium*, and several *Neuwiedia* species have wingless seeds with hard seed coats. Several members of Vanilleae, such as *Epistephium* and *Galeola*, have a hard seed coat over the embryo and a developed wing around the seed. Several *Neuwiedia* species have small seeds with sac-like appendages at either end. Most other orchids have a loose, rather papery seed coat around the embryo, which has a length that ranges from 0.15 mm to 6 mm. However, all of these orchid seeds are dry and lack an endosperm.

In this report, we documented a new orchid found on Danxia Mountain in Guangdong, China. The flower and seed structures of this new orchid are different from those of other known taxa in Orchidaceae. However, the phylogenetic clarification of this new orchid requires further molecular, morphological, and phytogeographic analyses.

## Results

### Morphological Analysis

**Figure 1:**
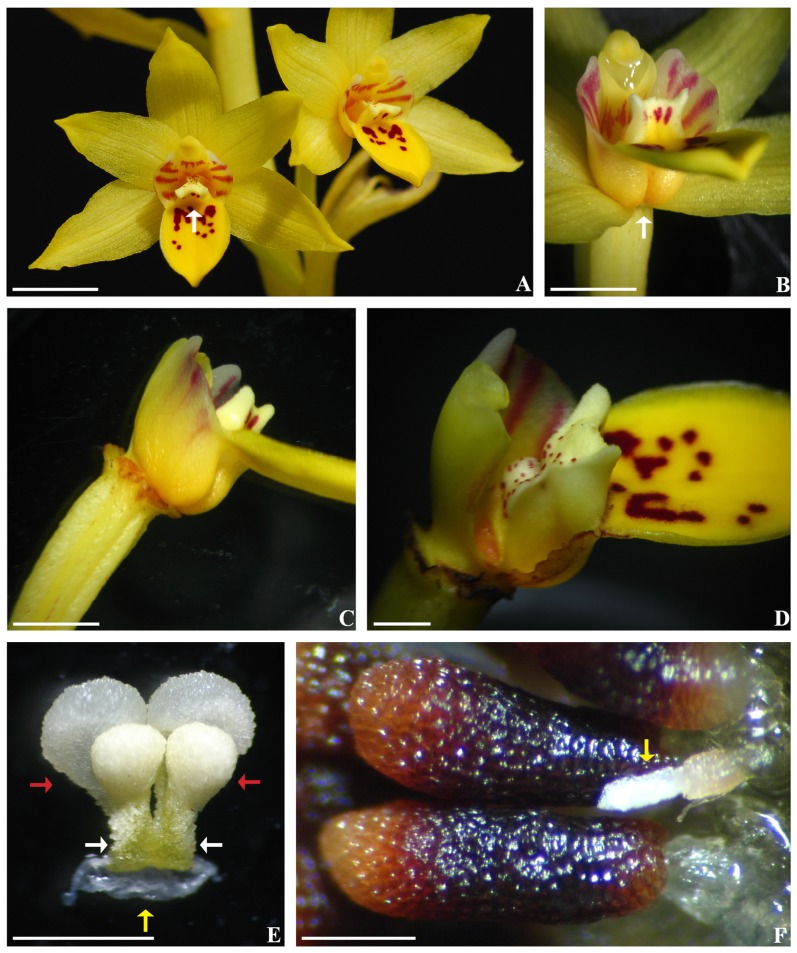
*Danxiaorchis singchiana* flowers and seeds. (A) Flowers with a Y-shaped appendage (arrow) on the labellum. Bar = 1 cm. (B) Labellum with two sacs (arrows) at the base. Bar = 4 mm. (C) Column and labellum, side view. Bar = 4 mm. (D). Appendage of the labellum, side view. Bar = 2 mm. (E) Pollinarium, front view, showing pollinia (red arrows), caudicles (white arrows), and viscidium (yellow arrow). Bar = 1 mm. (F) Mature Seeds, showing abortive seed (yellow arrow). Bar = 5 mm.

**Figure 2:**
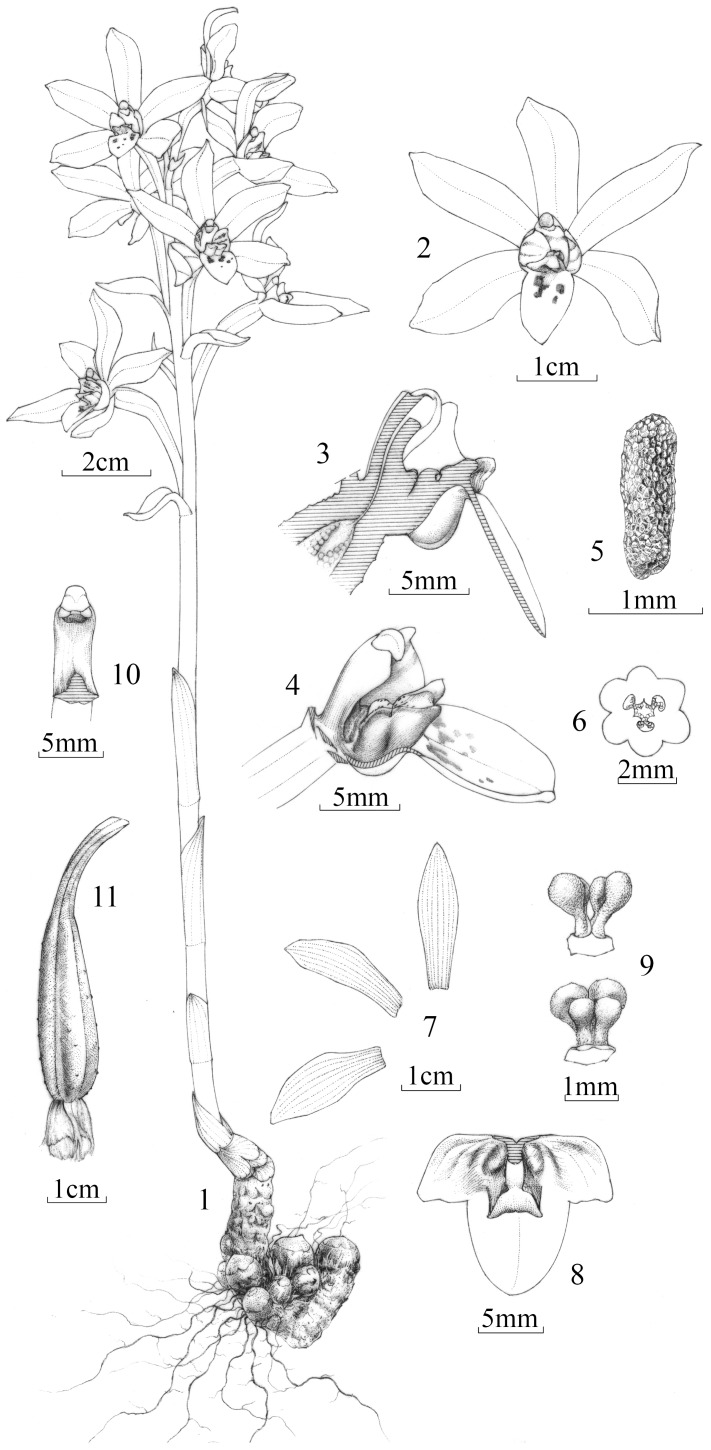
*Danxiaorchis singchiana*, J. W. Zhai, F. W. Xing et Z. J. Liu: (1) flowering plant; (2) flower, front view; (3) column and labellum, longitudinal section; (4) appendage of the labellum, side view; (5) seed; (6) ovary, cross section; (7) dorsal sepal, petal, and lateral sepal; (8) labellum, flattened; (9) pollinarium; (10) column, front view; (11) capsule.

The new orchid entity is restricted to the Danxia Mountain in northern Guangdong, China (Fig. S1). The Danxia region, known as the Danxia Landform, is famous for its topographic features. A detailed comparison between the newly discovered orchid and other members of Orchidaceae was conducted. The new plant is characterized by a labellum with two sacs at the base, an elongated column that has a terminal concave stigma and lacks conspicuous staminodes and rostellum, four sectile pollinia attached by two caudicles to a common large viscidium, and cylindrical, fleshy seeds. These features distinguish the new orchid from all other known orchids (Figs. 1, 2, and S2).

### Analyses of Phylogenetic Placement

**Figure 3:**
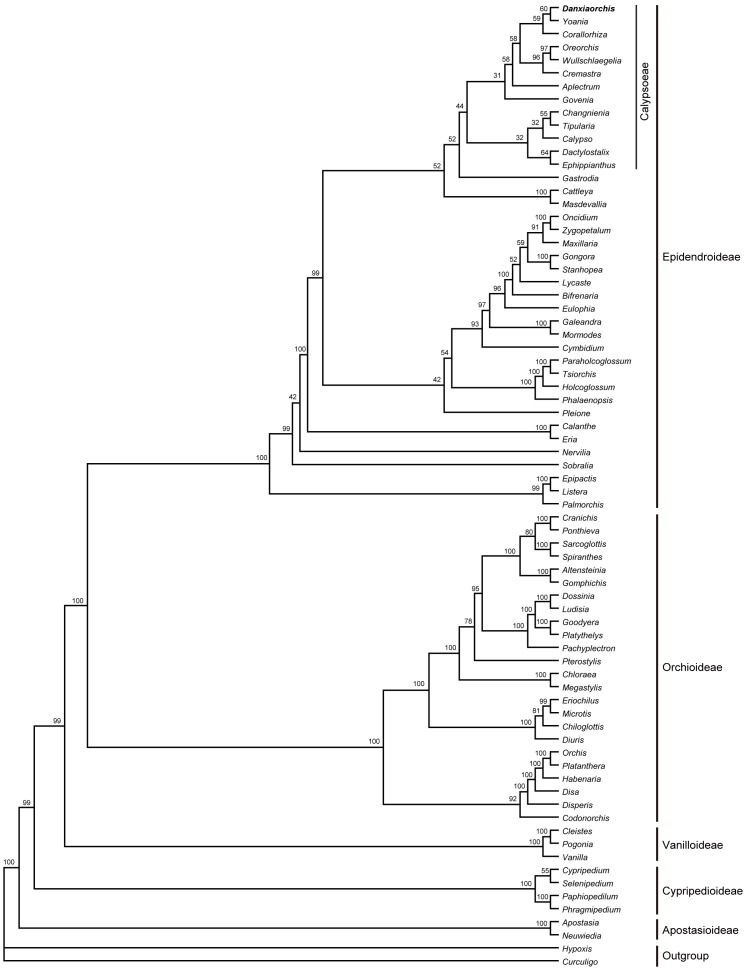
Bayesian consensus phylograms for the combined ITS, *mat*K, and *rbc*L datasets and 59 morphological character matrix, including 72 genera of Orchidaceae. The Bayesian PP (×100) is provided above the branches.

*Danxiaorchis singchiana* is morphologically related to the tribe Calypsoeae and, to a lesser degree, to the tribe Gastrodieae. Both of these tribes belong to the subfamily Epidendroideae. A detailed morphological character matrix (59 characters of 74 taxa) was integrated with a molecular matrix (3586 nucleotide sequences of the ITS, *mat*K, and *rbc*L genes of 74 genera) to classify the plant into an appropriate phylogenetic position (Figs. 3, S3, S4, and S5).

Bayesian inference (BI) phylogram showed the monophyly of the new orchid plant. Five clades were distinguished in Orchidaceae, with a posterior probability (PP) of over 99% (Fig. 3). Based on evolutionary sequences, the five clades correspond to the subfamilies Apostasioideae, Cypripedioideae, Vanilloideae, Orchidoideae, and Epidendroideae. Calypsoeae formed an independent lineage (PP = 52%) in the subfamily Epidendroideae, under which *Danxiaorchis* is included together with other genera of Calypsoeae. In the family-level BI phylogram of the combined ITS, *mat*K, and *rbc*L gene sequences, the Calypsoeae clade is divided into two subclades (PP = 95%, Fig. S4). The first subclade includes *Calypso*, *Tipularia*, and *Changnienia*, whereas the second subclade comprises eight genera, including *Danxiaorchis*, which is most closely related to *Yoania*, as confirmed by maximum parsimony (MP) analysis (Fig. S5).

### Phylogeny of Calypsoeae

#### Nuclear ITS sequence data analysis

The phylogenetic trees generated based on the ITS sequence data analysis clearly revealed the independence of the eight genera of this tribe. The BI phylogram with most of the clades received a strong support (PP>90%). *Danxiaorchis*, which forms a single clade with a PP of 99%, has been recognized as a natural genus within this tribe (Fig. S6). However, a relatively weak bootstrap and unstable topology is found in MP phylogram (Fig. S7).

#### Chloroplast sequence data analysis

Similarly, the 11 genera can be easily distinguished from the phylograms based on chloroplast sequence data analysis. The phylogenetic topologies generated by BI are approximately congruent with the ones by MP analysis (Figs. S8 and S9). The basal clade is independently composed of *Calypso*, *Tipularia*, and *Changnienia*. The next clade is *Govenia*, followed by a complex clade, which includes *Aplectrum*, *Cremastra*, *Danxiaorchis*, *Yoania*, *Wullschlaegelia*, and *Oreorchis*. *Corallorhiza* occupies the terminal positions in both MP and BI phylograms, although it is not well-supported intragenetically in the MP phylogram.

#### Combined analysis

In this study, ITS, *mat*K, and *rbc*L were combined into a single dataset. The strict consensus BI phylogram (Fig. S10) strongly supports the division of the Calypsoeae alliance (except *Dactylostalix* and *Ephippianthus*) into seven clades with eight subclades (PP = 100%, except for one with 77%). The first clade, which consists of the *Changnienia*, *Tipularia*, and *Calypso* subclades, is strongly supported as a sister to the outgroup clade, which consists of *Sobralia* and *Nervilia* (PP = 100%). The second clade, which has a single genus, *Govenia*, is strongly supported as a sister to the first clade (PP = 100%). The third clade is the *Aplectrum* genus, and the fourth is *Cremastra* (PP = 100%). The fifth clade contains the new genus *Danxiaorchis*, and its ally, *Yoania* (PP = 100%). The last two clades have weak support (PP = 77%). The sixth clade is comprised of *Wullschlaegelia* and *Oreorchis*. The seventh clade contains a single genus, *Corallorhiza*, which consists of 13 species that are further divided into two subclades. The results are in agreement with the results of the complex clades in the MP phylogram (Fig. S11).

**Figure 4:**
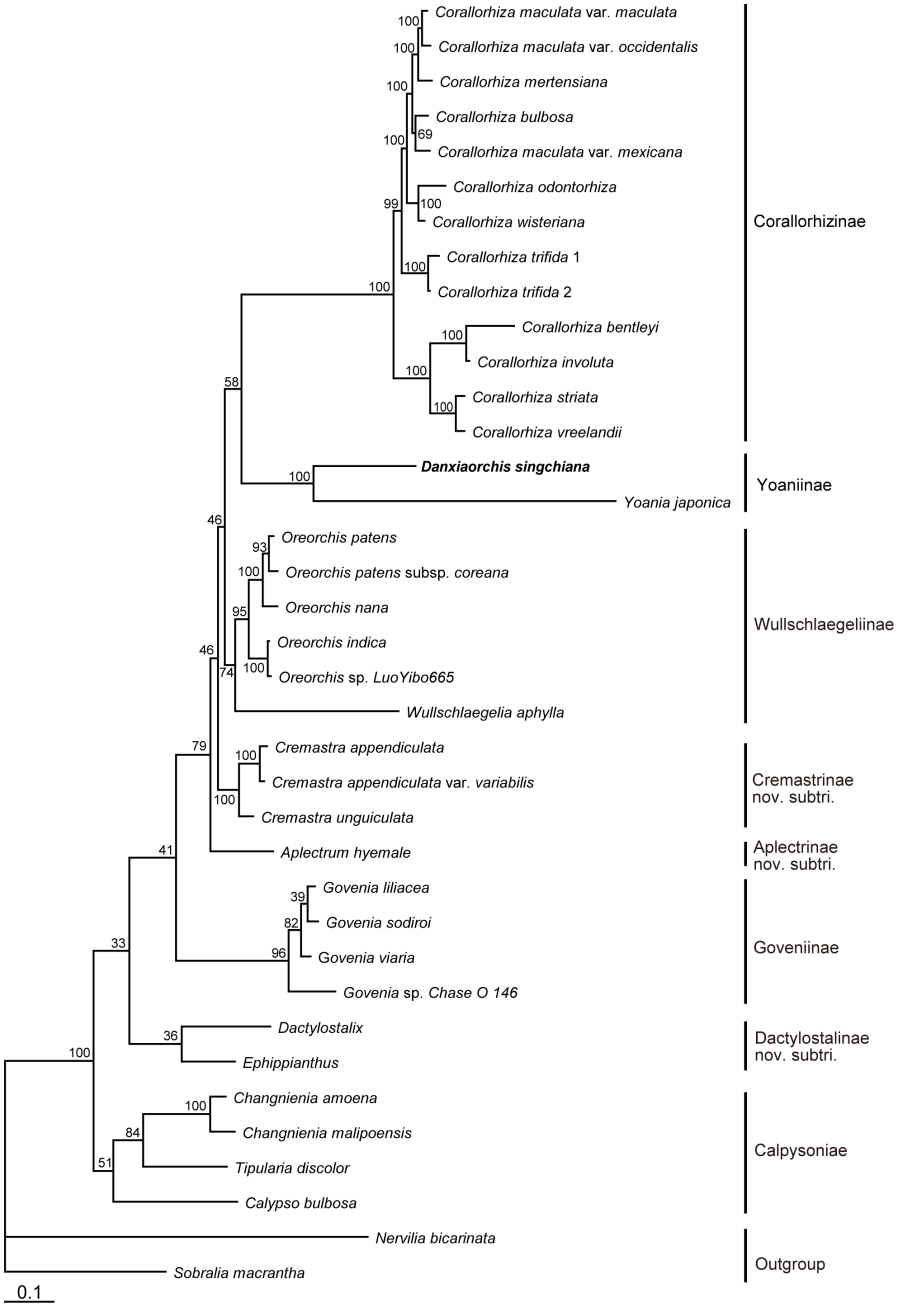
Bayesian consensus phylogram for the combined ITS, *mat*K, and *rbc*L datasets and 69 morphological character matrix, including 35 taxa of Calypsoeae. Bayesian PP (×100) is given above the branches.

**Figure 5:**
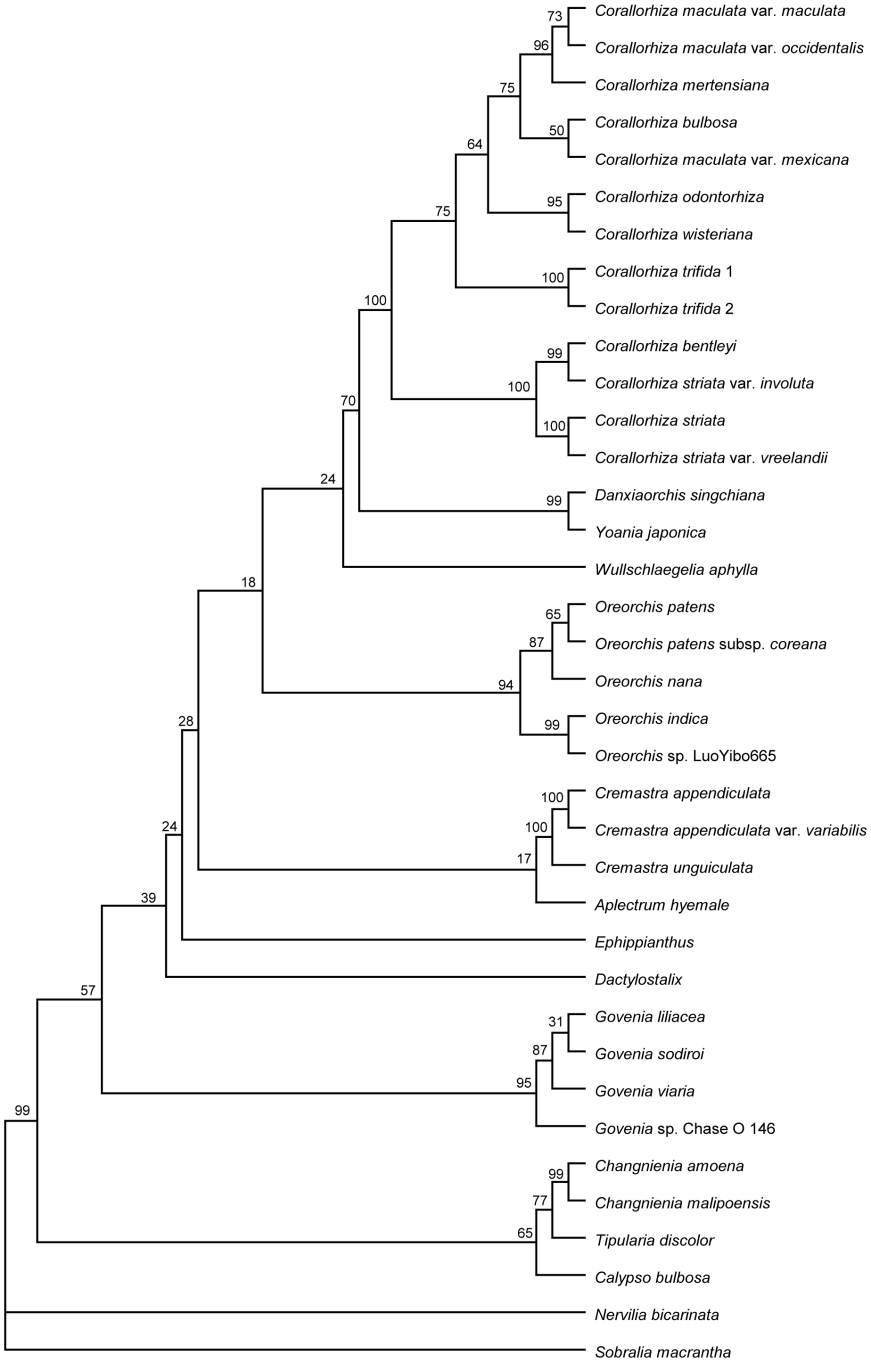
Strict consensus phylogram of most parsimonious phylograms based on the ITS, *mat*K, and *rbc*L datasets and 69 morphological character matrix, including 35 taxa of Calypsoeae. The bootstrap values of the MP analysis are given above the branches. Tree length = 3023 steps; CI = 0.7291; RI = 0.8167.

The sequence data of each species and the morphological characters of each genus were combined into a single dataset (*Dactylostalix* and *Ephippianthus* having morphological characters only). The strict consensus BI phylogram supports the division of the 13 genera of Calypsoeae into four clades with 13 subclades, which is in agreement with the results of the combined sequence data analysis. These results show that the genera *Dactylostalix* and *Ephippianthus* belong to a single clade near the *Govenia* and *Calypso* clades (Figs. 4 and 5).

## Discussion

### Morphological Analysis

This study is the first to report an orchid with a bisaccate labellum and cylindrical, fleshy seeds. This orchid is difficult to classify in any known subtribe or tribe within Orchidaceae. Although the new orchid has a particular similarity to *Gastrodia* and its allies in terms of pollinium structure, the pollinarium of *Gastrodia* lacks distinct caudicles and viscidium like the new orchid does. This new orchid sharply differs from *Satyrium* and *Corybas* except for the two elongate or saccate spurs at the base of their labellum [9]. *Danxiaorchis* has cylindrical (1.5 mm×0.5 mm) and fleshy seeds, which is similar to the seeds of underground orchid species of *Rhizanthella* [10]. However they’re distinct from *Danxiaorchis* by its underground habitat, absence of roots, fleshy overlapping bracts, small flowers and minute seeds. Thus, this genus is distinguishable from all other genera of orchids.

### Family-level Analysis

The results of our analyses are in agreement with those obtained by previous researchers [5], [9], [11], [12] and support the relationships among the subfamilies Apostasioideae, Vanilloideae, Cypripedioideae, Orchidoideae, and Epidendroideae. In addition, a more precise phylogenetic tree was obtained in the present study at higher categories. This finding may be attributed in part to the utilization of a more diversified nuclear genetic marker, ITS, the application of multiple genetic markers, and the integration of morphological and molecular characters.

### Calypsoeae-level Analysis

Dressler [5] and Pridgeon et al. [ed.] [13] investigated the tribe Calypsoeae but did not propose any subtribe. Dressler defined Calypsoeae as comprising nine genera, namely, *Aplectrum*, *Calypso*, *Corallorhiza*, *Cremastra*, *Dactylostalix*, *Ephippianthus*, *Oreorchis*, *Tipularia*, and *Yoania*. Pridgeon et al. [ed.] [13] added *Wullschlaegelia* and *Govenia* to this tribe, although Dressler had placed the former in Gastrodiinae and the latter in Cymbidieae [5], both at subtribal rank. Meanwhile, Chen et al. [14] treated these genera as members of the tribe Epidendreae, with the following subtribes in China: subtribe Yoaniinae with the genus *Yoania*; subtribe Calypsoinae with the genera *Oreorchis*, *Cremastra*, *Tipularia*, *Calypso*, and *Changnienia*; and subtribe Corallorhizinae with the genus *Corallorhiza*. However, all of these classification systems were based only on morphological evidence.

*Govenia* and *Corallorhiza* are both monophyletic genera (PP = 100%), with the former genus having a relatively anomalous distribution in the tribe Calypsoeae [13]. Thus, their subtribal rank, the subtribes Goveniinae [5] and Corallorhizinae [14], is maintained. The phylogenetic placement and the infrageneric relationships of Corallorhizinae are very similar to those studied by Freudenstein et al. [15] *Corallorhiza* should be divided into two subclades. The first subclade contains *C. striata* Lindl., *C. bentleyi* Freudenst., *C. involuta* Greenm., and *C. vreelandii* Rydb. The species of this subclade possess three-veined perianth segments and a thickened labellum that are fused at the base. The second subclade contains the rest of the species in this genus. They possess a thin-textured labellum, and some species, such as *C. trifida* Châtel. and *C. odontorhiza* (Willd.) Nutt. are autogamous [15]–[18].

*Aplectrum* and *Wullschlaegelia* are composed of a few species that are distributed from North America to tropical South America. For *Wullschlaegelia*, a monotypic subtribe Wullschlaegeliinae was established in 1990 [5]. *Cremastra* and *Oreorchis* are only found in Asia [19], [20]. *Yoania* is most closely related to *Danxiaorchis* and they share the same habitat. However, *Danxiaorchis* can be distinguished from *Yoania* by its rooted rhizome, bisaccate labellum, Y-shaped appendages, and caudicles [21].

The Danxia Mountain located in northern Guangdong, where *Danxiaorchis* grows, was formed approximately 6 million years ago [22]. The unique geological conditions and the relative environmental isolation of the Danxia Mountain might have favored the speciation of new taxa, such as *Oberonioides microtatantha* (Schltr.) Szlach. [14], *Firmiana danxiaensi*s H. H. Hsue et H. S. Kiu [23], and *Lyonia danxiaensis* Miau et W. Q. Liu [24].

Two distantly related genera, *Dactylostalix* and *Ephippianthus* [13], are distributed along the Sakhalin Peninsula in northern Japan and the Kuriles. Unfortunately, materials from these two genera could not be obtained. However, in our study, these two genera formed a sister clade with the *Calypso* and *Govenia* clades based on their morphological characters.

### Conclusion

The Danxia orchid has several distinct features. Based on results obtained by applying phylogenetic methods to a matrix of morphological and molecular characters, the Danxia orchid can be treated as a new genus of Calypsoeae (subfamily Epidendroideae). Analysis of the combined datasets using maximum likelihood methods revealed strong evidence that Calypsoeae is a monophyletic tribe consisting of eight well-supported clades with 13 subclades, which are all in agreement with the phytogeography of Calypsoeae.

The Danxia orchid represents an independent lineage under the tribe Calypsoeae of the subfamily Epidendroideae. This lineage should be treated as a new genus parallel to *Yoania* under the subtribe Yoaniinae. The new classification should be as follows:

Subfamily: Epidendroideae

Tribe: Calypsoeae

**Subtribe: Yoaniinae** Szlach.

**Danxiaorchis singchiana** J. W. Zhai, F. W. Xing, and Z. J. Liu gen. et sp. nov. (Figs. 1, 2, and S2) [*Danxiaorchis*, urn:lsid:ipni.org:names: 77124908-1; *D. singchiana*, urn:lsid:ipni.org:names: 77124909-1].

- **Etymology:** The generic name alludes to Danxia, the name of the locality where it was found. The Danxia Mountain is famous for its topographic feature, the Danxia Landform. The Greek name for orchid, orchis, is then incorporated. Thus, *Danxiaorchis* refers to an orchid growing on the Danxia Mountain. The specific epithet *singchiana* is dedicated to the Chinese professor Sing-Chi Chen, a famous, internationally renowned orchidologist.
- **Type**: Guangdong, Renhua, Danxiashan, in a forest, alt. 125 m, 2011.5.31. *J. W. Zhai*, *5481* (holotype, NOCC; isotype: IBSC).
- **Diagnosis**: *The new remarkable genus is distinct from all known orchid genera by it possesses a labellum with a large Y-shaped callus and two sacs at the base, and cylindrical, fleshy seeds.*
- **Description**: Holomycotrophic plants 21 to 40 cm tall; rhizome tuberous, fleshy, cylindrical, 5 to 6 cm long, 0.6 to 1.8 cm thick, shortly branched, rooting. Scape erect, terete, pale red-brown, slightly tinged with green-yellow, 3- to 4-sheathed; sheaths, cylindrical, clasping stem, membranous, 2.3 to 3.5 cm long; raceme 5 to 8.5 cm long, 2- to 13-flowered; floral bracts oblong-lanceolate, 1.5 to 2.3 cm long; pedicel and ovary 2.2 to 4.6 cm long, glabrous; sepals and petals pale yellow; lip yellow, with pale purple-red stripes on side-lobes and purple-red spots on mid-lobe; dorsal sepals narrowly elliptic, 1.8 to 2.6 cm × 6 to 9 mm, acute; lateral sepals obovate-elliptic, 2 to 2.3 cm×7 to 9 mm, acute; petals narrowly elliptic, 2 to 2.2 cm×6.5 to 7.5 mm, acute; labellum 3-lobed; side-lobes erect, slightly clasping the column, subsquare, up to 5 mm long and 5.5 mm wide; mid-lobe oblong, 7 to 8 mm×5 to 8 mm, apex rounded-obtuse; labellum with two sacs at the base and a Y-shaped fleshy appendage centrally; appendage extending from the base of disc to the base of mid-lobe, 1.3 to 1.5 mm tall; column semi-terete, 5 to 7 mm long, footless; stigma concave, terminal; anther cap ellipsoid; pollinia four, in two pairs, subobovoid-globose, granular-farinaceous, composed of friable massulae, each pair containing two pollinia unequal in size with a thick caudicle attached to a common subsquare viscidium. Capsule fusiform, 3 to 4.2 cm long, 0.8 to 1.2 cm thick. Seeds cylindrical, 1.5×0.5 mm, fleshy. Fl. April–May. Fr. May–June.

## Materials and Methods

### Materials

The locations of the field studies are neither private lands nor protected areas, but are controlled by the State Forestry Administration of China, to which our institution is affiliated. The State Forestry Administration authorized us to conduct scientific observations or tests in the regions it controls.A valid permit was also obtained for testing the genes of *Danxiaorchis.*

A total of 74 genera were analyzed in the family-level study. Two genera, *Hypoxis* and *Curculigo*, were selected as outgroups. Three genetic markers (ITS, *mat*K, and *rbc*L) of *Danxiaorchis*, *Corallorhiza*, *Cremastra*, *Oreorchis*, and *Yoania* were analyzed. The gene sequences of the other 61 genera were accessed from GenBank (Table S1). *Danxiaorchis singchiana* was collected from the Danxia Mountain in northern Guangdong, China (25°N, 113°E).

A total of 34 species (or subspecies or varieties) and 35 individuals of 13 genera were included in the tribe-level analysis, wherein *Sobralia* and *Nervilia* were selected as outgroups. The ITS, *mat*K, and *rbc*L gene sequences of *Danxiaorchis singchiana*, *Corallorhiza trifida*, *Changnienia malipoensis*, *Cremastra appendiculata*, *Yoania japonica*, *Oreorchis indica*, and *O. nana* were applied in the same way as that in the family-level study. The other sequences were accessed from GenBank (Table S2).

*Corallorhiza trifida* and *Oreorchis nana* were collected from Huanglong in Sichuan Province. *Cremastra appendiculata* was cultivated in a nursery in Shenzhen, whereas *Yoania japonica* was obtained from the herbarium of The Orchid Conservation and Research Center of Shenzhen (NOCC, Z. J. Liu 6241).

*Danxiaorchis singchiana* was collected between April 2012 and May 2012 from its habitat in northern Guangdong, China. Several individual plants with young fruits were cultivated in our nursery in Shenzhen for mature fruits and seeds. Fresh flowers, especially the pollinaria, were examined using a stereoscope (Guiguang XTL-500, China). Colour photographs, black-white drawings, and descriptions were catelogued at the time. Molecular experiments were performed at the Shenzhen Key Laboratory for Orchid Conservation and Utilization of The Orchid Conservation and Research Center of Shenzhen.

All material for morphological and molecular examinations was kept in FAA (55% alcohol: glacial acetic acid: formalin at a ratio of 95∶5∶5) and allochroic silica gel.

### Methods

#### Amplification and sequencing

Total DNA was extracted from fresh material, silica gel-dried plant tissue, or herbarium specimens using a modified hexadecyl trimethyl ammonium bromide method [25].

The amplification reaction included total DNA, primers, Mighty Amp buffer version 2.0, and Mighty Amp DNA polymerase (Takara Bio). The polymerase chain reaction (PCR) profile consisted of an initial 2 min pre-melt stage at 98°C; 35 cycles of 20 s at 98°C (denaturation), 20 s at 45°C to 55°C (annealing temperature was determined by the requirements of the primer), and 50 s to 90 s at 68°C (extension time was determined by the length of the target DNA region); and a final extension of 6 min to 8 min at 68°C.

Amplification of the ITS, *mat*K, and *rbc*L regions was separately performed using the primer pairs ITS A and ITS B, *mat*K-19F and *trn*K-2R, and *rbc*L [26]–[28]. Other *mat*K and *rbc*L primer sets were also amplified (Table S3).

The PCR products were run on 1.5% agarose gels to check the amplified DNA quality. Gels with target products were excised, purified using DNA gel extraction kits (OMEGA BIO-TEK, USA), and sequenced by Invitrogen (Shanghai).

#### Sequence editing and assembling

The forward and reverse sequences as well as electropherograms were edited and assembled using DNASTAR (http://www.dnastar.com/). The DNA sequences were aligned using MEGA5.05 using Muscle method [29] and then manually adjustments were made for inserting gaps to improve the alignments [30]. The aligned sequences are available from the corresponding authors upon request.

#### Morphological analyses

A matrix, which consists of 59 morphological characters of 74 taxa in the family-level analysis (Morphological Character Codes S1 and Table S4) and 69 morphological characters of 35 taxa in the tribe-level analysis (Table S5), was constructed to explore the phylogenetic positions of the *Danxiaorchis* alliance by morphological classification.

#### Data analyses

Maximum Parsimony (MP) analyses were performed usingPAUP* version 4.0b10 [31]. All characters were equally weighed and unordered. The test settings included 1,000 replications of random addition sequence and heuristic search with tree bisection and reconnection branch swapping. Tree length, consistency indices (CI), and retention indices (RI) are shown in Table S6. BI analysis was performed using MrBayes3.1.2 [32]. The best-fit model for each dataset was selected using Modeltest 3.7. The model for the combined ITS, *mat*K, and *rbc*L datasets was also based on the best-fit model for each individual dataset (Tables S7 and S8). The following settings were applied: sampling frequency = 100; temp = 0.1; burn-in = 10,000; and the number of Markov Chain Monte Carlo generations = 4,000,000. The first 10,000 trees were discarded as burn-in. A majority-rule consensus phylogram was constructed based on the phylograms sampled after the 1,000,000^th^ generation.

### Nomenclature Acts

The electronic version of this article in Portable Document Format (PDF) in a work with an ISSN or ISBN will represent a published work according to the International Code of Nomenclature for algae, fungi, and plants, and hence the new names contained in the electronic publication of a PLOS ONE article are effectively published under that Code from the electronic edition alone, so there is no longer any need to provide printed copies.

In addition, new names contained in this work have been submitted to IPNI, from where they will be made available to the Global Names Index. The IPNI LSIDs can be resolved and the associated information viewed through any standard web browser by appending the LSID contained in this publication to the prefix http://ipni.org/. The online version of this work is archived and available from the following digital repositories: PubMed Central, LOCKSS.

## Supporting Information

Figure S1 ***Danxiaorchis*** **location.** Map showing the *Danxiaorchis* locality (star) in the Danxia Landform in northern Guangdong Province, China. The inset map shows the location of Guangdong Province in southern China.
(TIF)

Figure S2 ***Danxiaorchis singchiana*** **.** (A) Flowering plants in their habitat. Bar = 4 cm; (B) Inflorescence. Bar = 1.5 cm; (C) Fruiting plant. Bar = 2 cm; (D) Tuberous rhizome. Bar = 6 mm.
(TIF)

Figure S3 **Strict consensus phylogram of most parsimonious phylograms based on the combined ITS,** ***mat*** **K, and** ***rbc*** **L datasets and a matrix composed of 59 morphological characters of 72 Orchidaceae genera.** Bootstrap values of the MP analysis are indicated above the branches. Tree length = 18095 steps; CI = 0.2354; RI = 0.5600.
(TIF)

Figure S4 **Bayesian consensus phylogram for the combined ITS,** ***mat*** **K, and** ***rbc*** **L datasets, including 71 genera of Orchidaceae.** Bayesian PP (×100) is indicated above the branches.
(TIF)

Figure S5 **Strict consensus phylogram of most parsimonious phylograms based on the combined ITS,** ***mat*** **K, and** ***rbc*** **L datasets, including 71 genera of Orchidaceae.** Bootstrap values for the MP analysis are indicated above the branches. Tree length = 10188 steps; CI = 0.3248; RI = 0.5857.
(TIF)

Figure S6 **Bayesian consensus phylogram for the combined ITS datasets, including 26 taxa of Calypsoeae.** Bayesian PP (×100) is indicated above the branches.
(TIF)

Figure S7 **Strict consensus phylogram of most parsimonious phylograms based on ITS datasets, including 26 taxa of Calypsoeae.** The bootstrap values of the MP analysis are indicated above the branches. Tree length = 444 steps; CI = 0.8153; RI = 0.6641.
(TIF)

Figure S8 **Bayesian consensus phylogram for the combined** ***mat*** **K and** ***rbc*** **L datasets, including 32 taxa of Calypsoeae.** Bayesian PP (×100) is indicated above the branches.
(TIF)

Figure S9 **Strict consensus phylogram of most parsimonious phylograms based on the combined** ***mat*** **K and** ***rbc*** **L datasets, including 32 taxa of Calypsoeae.** The bootstrap values of the MP analysis are indicated above the branches. Tree length = 931 steps; CI = 0.8217; RI = 0.8903.
(TIF)

Figure S10 **Bayesian consensus phylogram for the combined ITS,** ***mat*** **K, and** ***rbc*** **L datasets, including 33 taxa of Calypsoeae.** Bayesian PP (×100) is indicated above the branches.
(TIF)

Figure S11 **Strict consensus phylogram of most parsimonious phylograms based on the ITS,** ***mat*** **K, and** ***rbc*** **L datasets, including 33 taxa of Calypsoeae.** The bootstrap values of the MP analysis are indicated above the branches. Tree length = 1505 steps; CI = 0.7980; RI = 0.8550.
(TIF)

Table S1 **Samples used in Orchidaceae gene sequencing and their information.**
(DOC)

Table S2 **Samples used in Calypsoeae gene sequencing and their information.**
(DOC)

Table S3 **Primers used in this study.**
(DOC)

Table S4 **Morphological data matrix for the phylogenetic analysis.**
(DOC)

Table S5 **Morphological data matrix for the tribe-level phylogenetic analysis.**
(DOC)

Table S6 **Statistics from the analyses of various datasets.**
(DOC)

Table S7 **Best-fit model and parameter for each Orchidaceae dataset.**
(DOC)

Table S8 **Best-fit model and parameter for each Calypsoeae dataset.**
(DOC)

Morphological Character Codes S1 (DOC)
