# Supplementary figures and images for: A New Orchid Genus, Danxiaorchis, and Phylogenetic Analysis of the Tribe Calypsoeae
Source: PLoS One. 2013 Apr 4;8(4):e60371. doi: 10.1371/journal.pone.0060371 (PMC3617198; doi:10.1371/journal.pone.0060371)

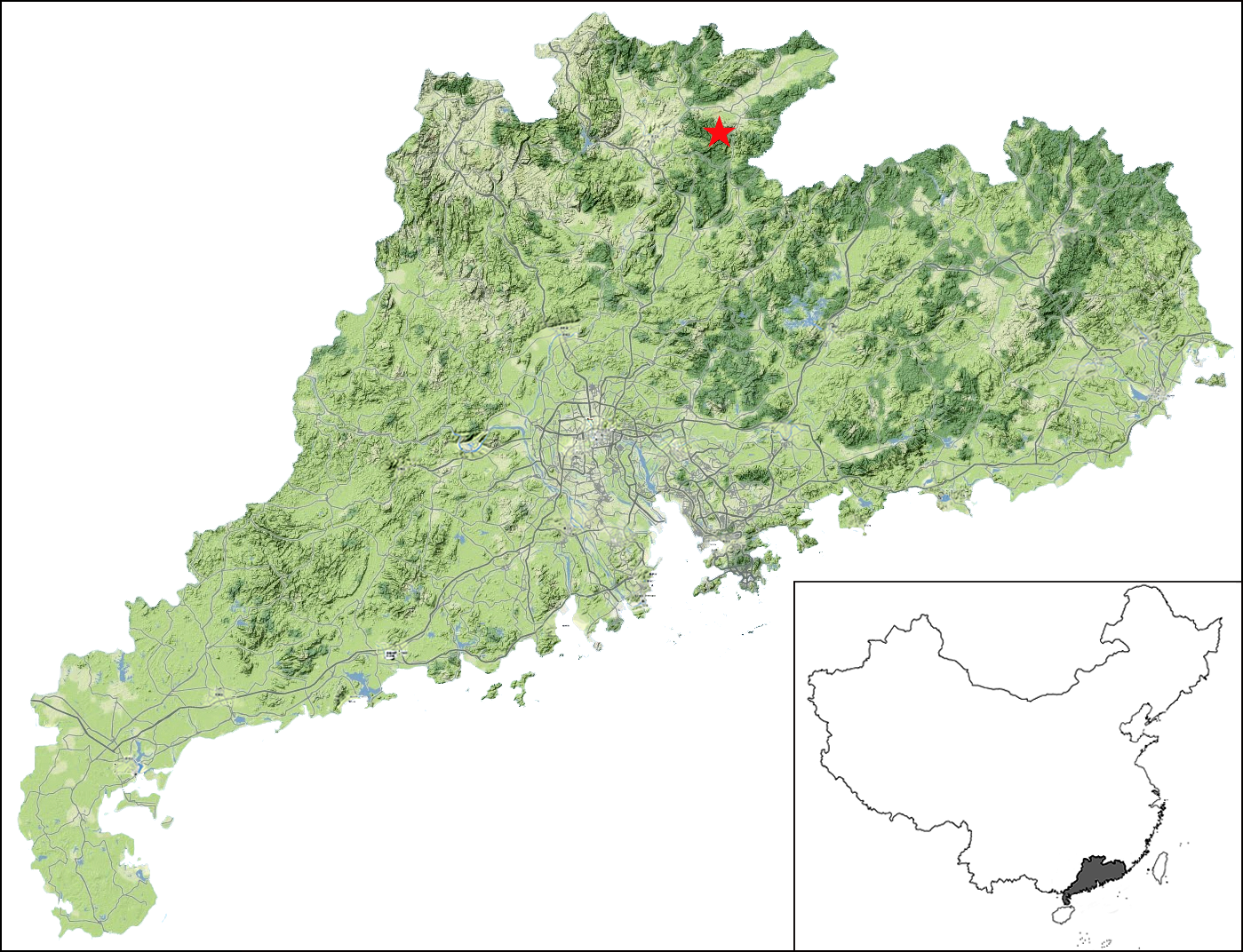

Supplement: Figure S1 — Danxiaorchis location. Map showing the Danxiaorchis locality (star) in the Danxia Landform in northern Guangdong Province, China. The inset map shows the location of Guangdong Province in southern China. (TIF) [file pone.0060371.s001.tif]

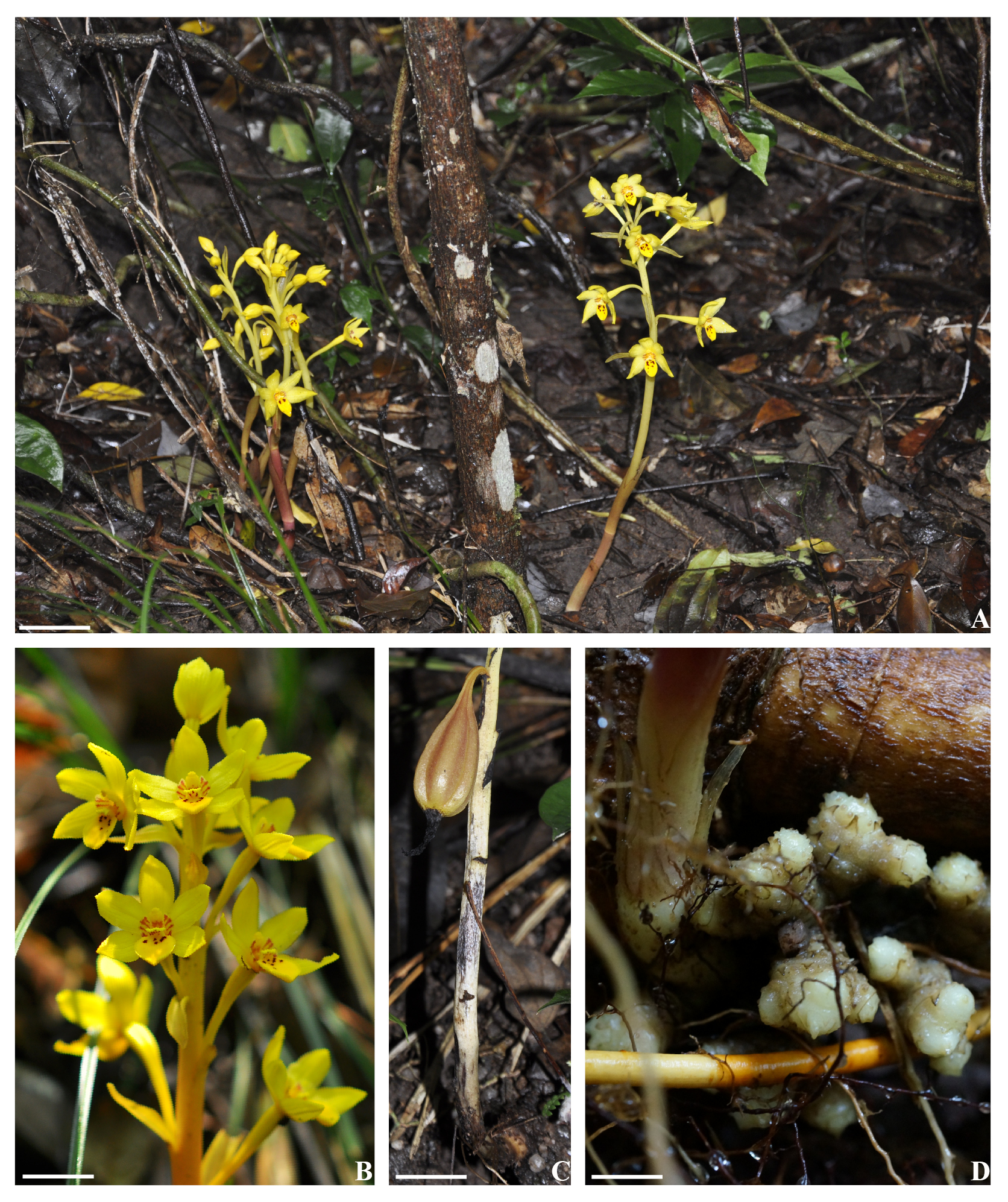

Supplement: Figure S2 — Danxiaorchis singchiana . (A) Flowering plants in their habitat. Bar = 4 cm; (B) Inflorescence. Bar = 1.5 cm; (C) Fruiting plant. Bar = 2 cm; (D) Tuberous rhizome. Bar = 6 mm. (TIF) [file pone.0060371.s002.tif]

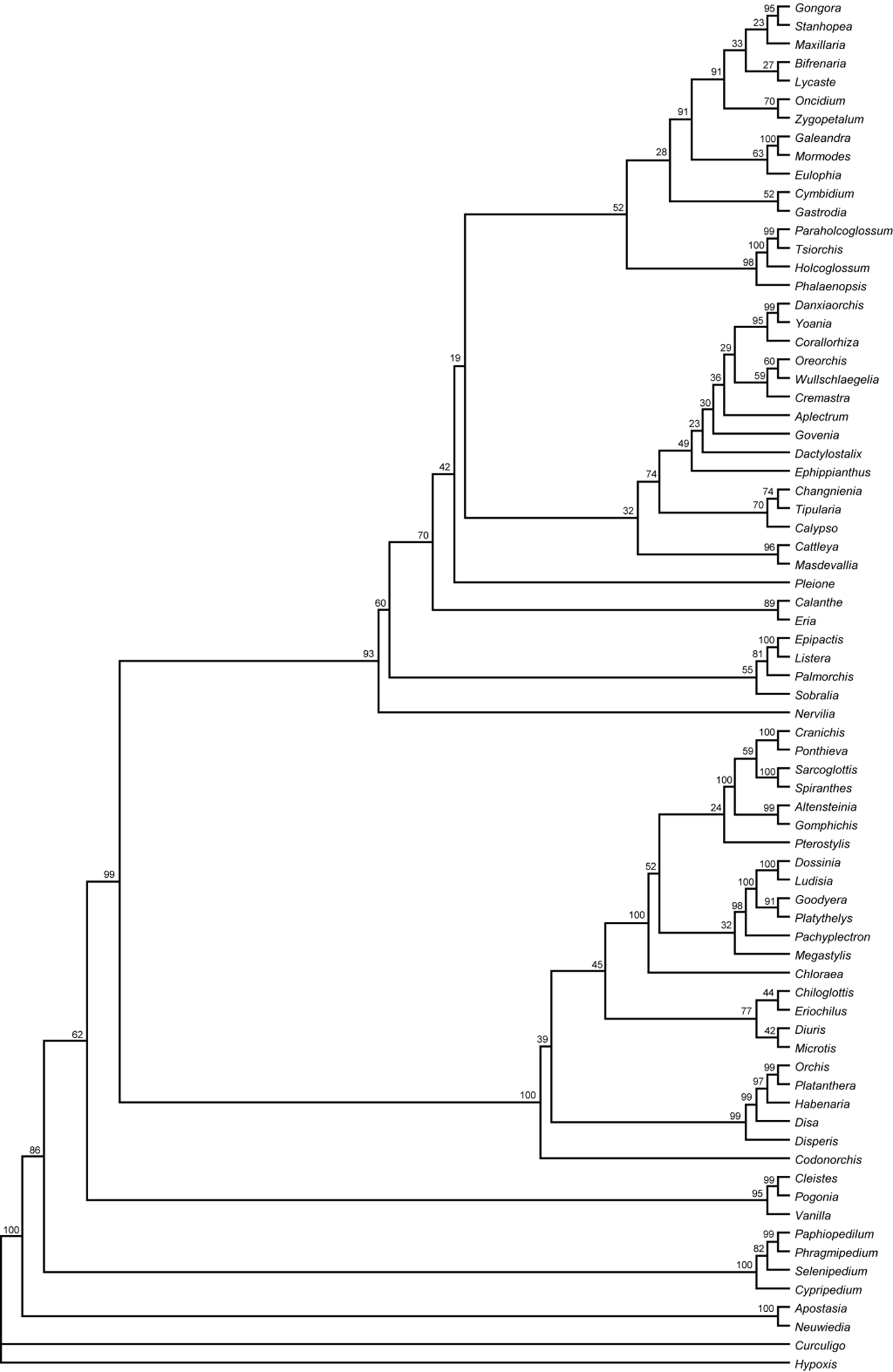

Supplement: Figure S3 — Strict consensus phylogram of most parsimonious phylograms based on the combined ITS, mat K, and rbc L datasets and a matrix composed of 59 morphological characters of 72 Orchidaceae genera. Bootstrap values of the MP analysis are indicated above the branches. Tree length = 18095 steps; CI = 0.2354; RI = 0.5600. (TIF) [file pone.0060371.s003.tif]

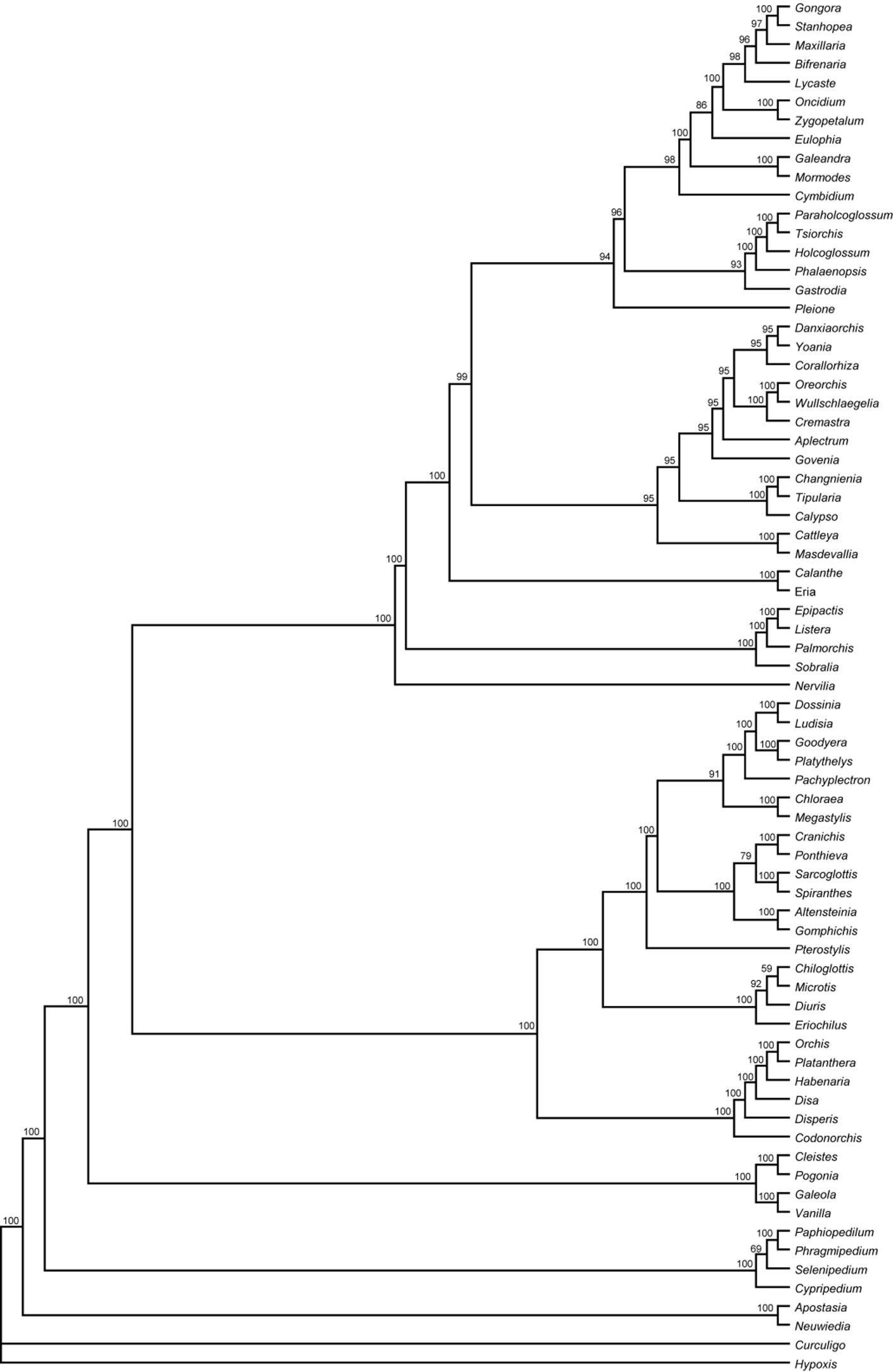

Supplement: Figure S4 — Bayesian consensus phylogram for the combined ITS, mat K, and rbc L datasets, including 71 genera of Orchidaceae. Bayesian PP (×100) is indicated above the branches. (TIF) [file pone.0060371.s004.tif]

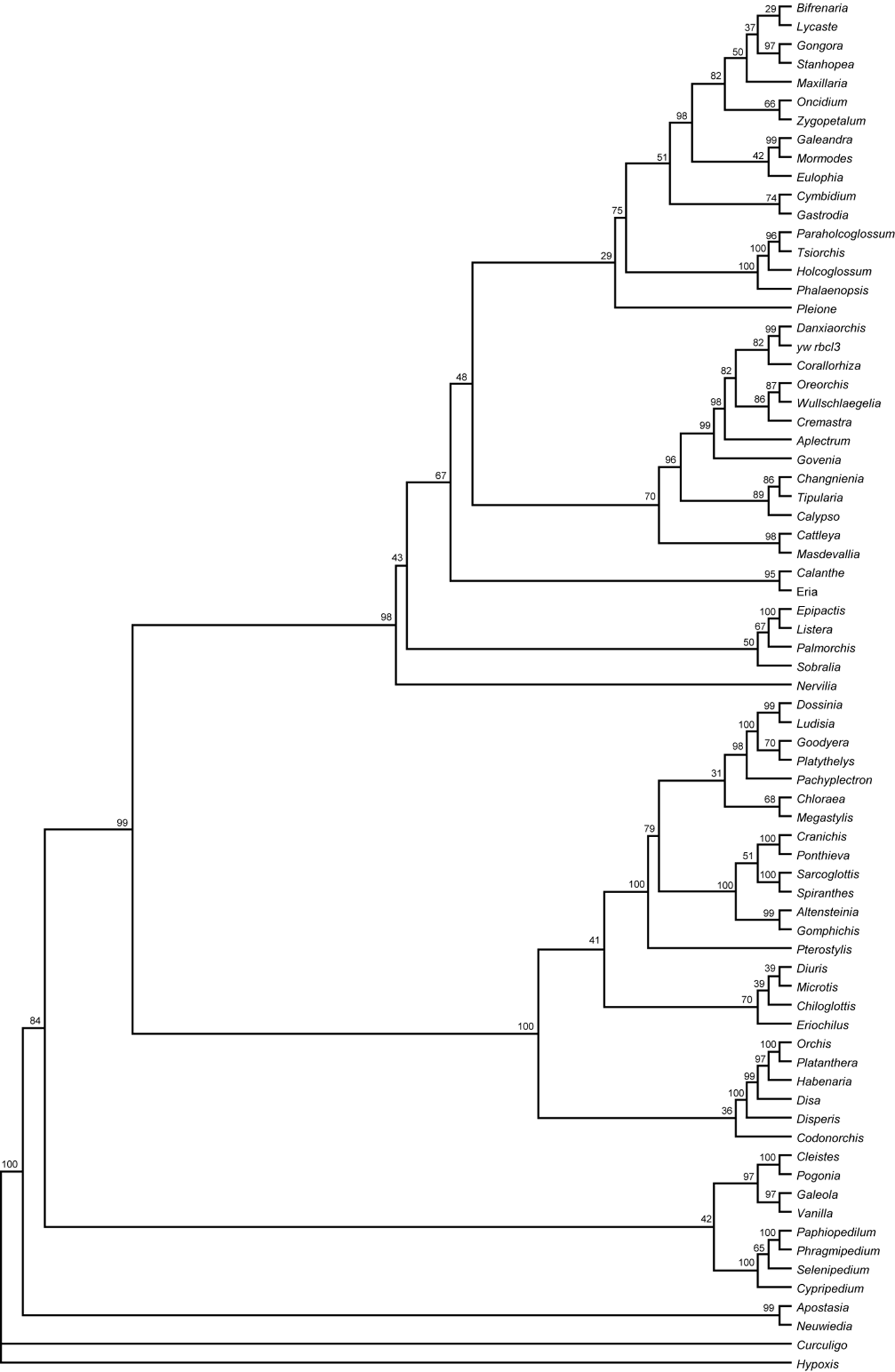

Supplement: Figure S5 — Strict consensus phylogram of most parsimonious phylograms based on the combined ITS, mat K, and rbc L datasets, including 71 genera of Orchidaceae. Bootstrap values for the MP analysis are indicated above the branches. Tree length = 10188 steps; CI = 0.3248; RI = 0.5857. (TIF) [file pone.0060371.s005.tif]

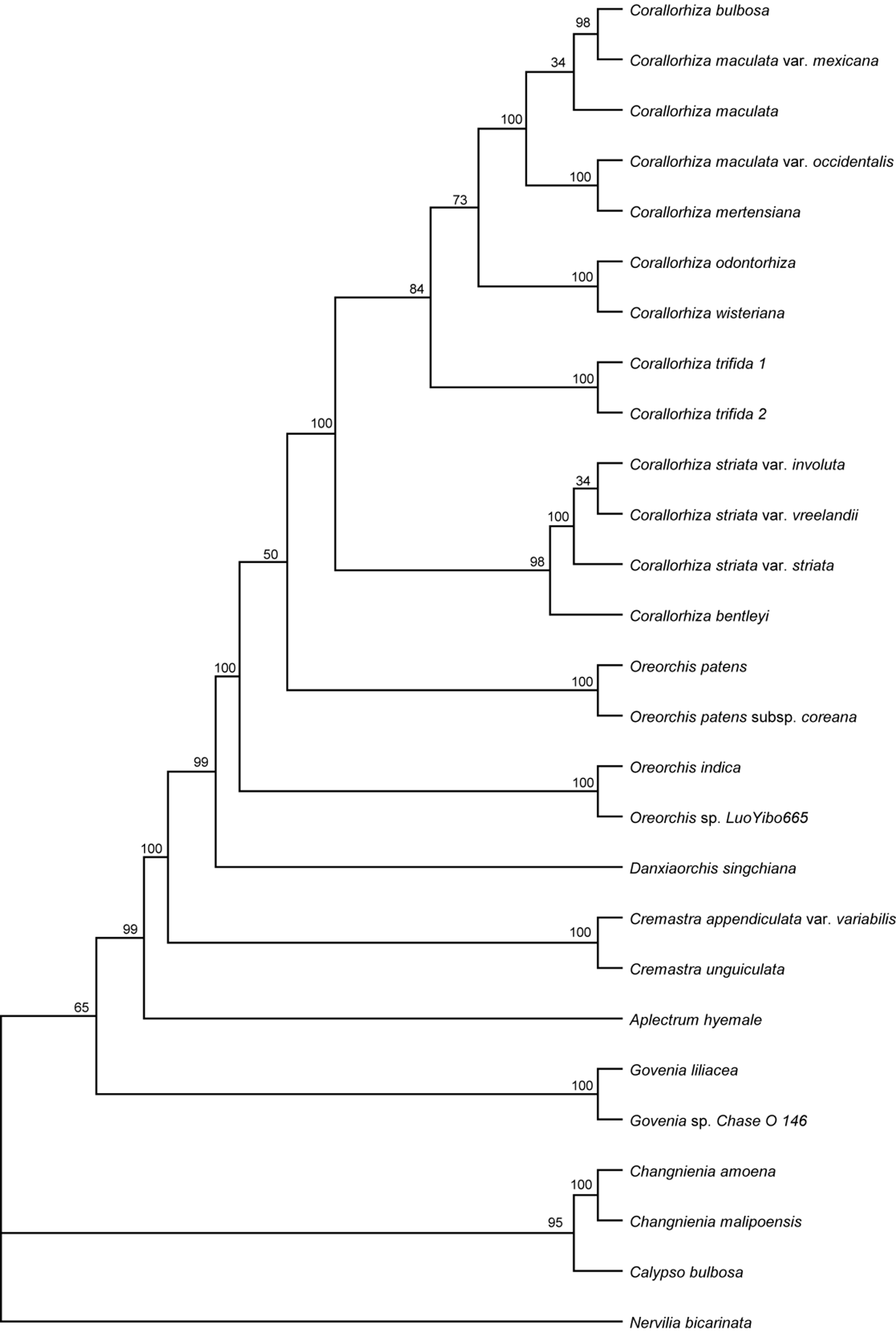

Supplement: Figure S6 — Bayesian consensus phylogram for the combined ITS datasets, including 26 taxa of Calypsoeae. Bayesian PP (×100) is indicated above the branches. (TIF) [file pone.0060371.s006.tif]

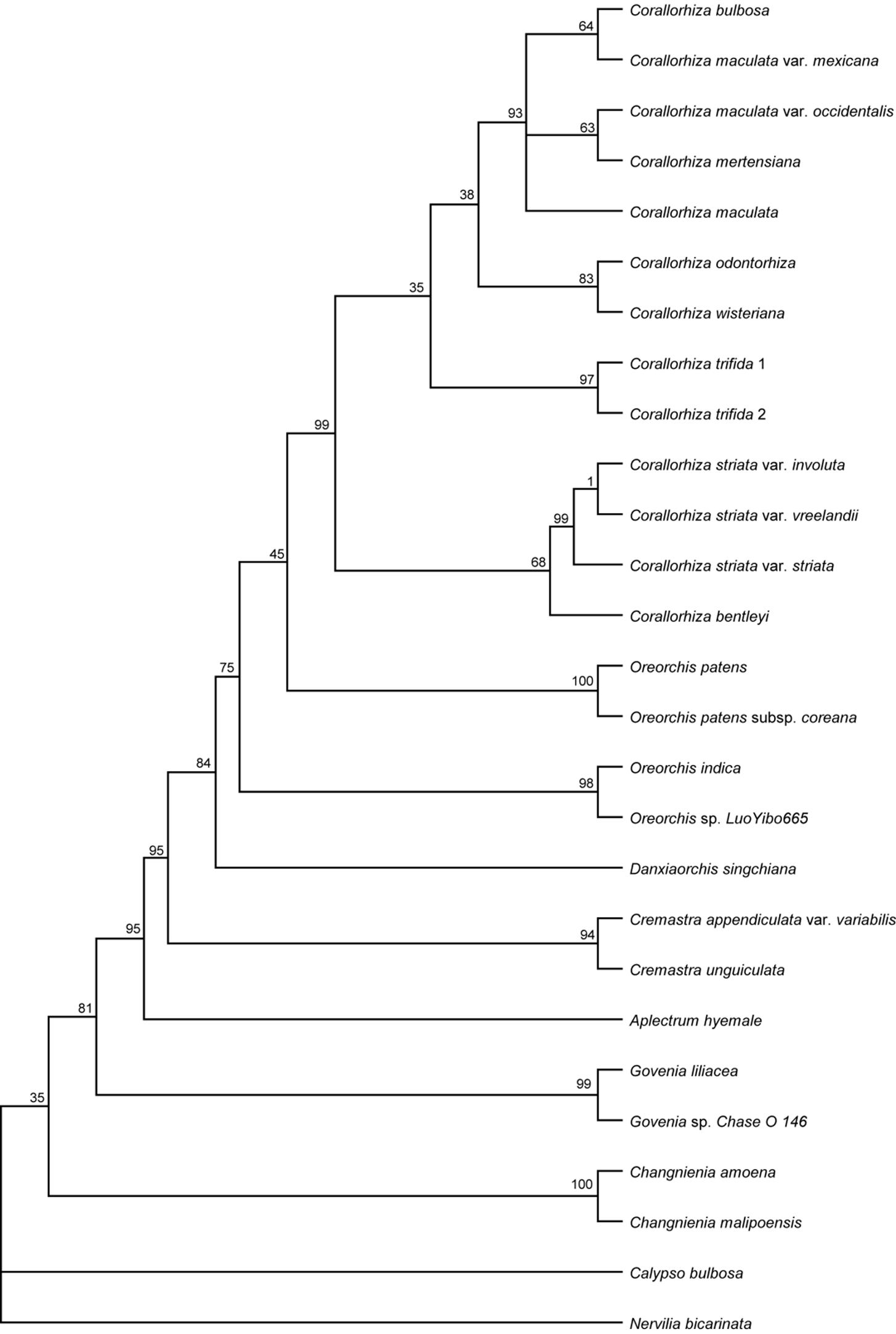

Supplement: Figure S7 — Strict consensus phylogram of most parsimonious phylograms based on ITS datasets, including 26 taxa of Calypsoeae. The bootstrap values of the MP analysis are indicated above the branches. Tree length = 444 steps; CI = 0.8153; RI = 0.6641. (TIF) [file pone.0060371.s007.tif]

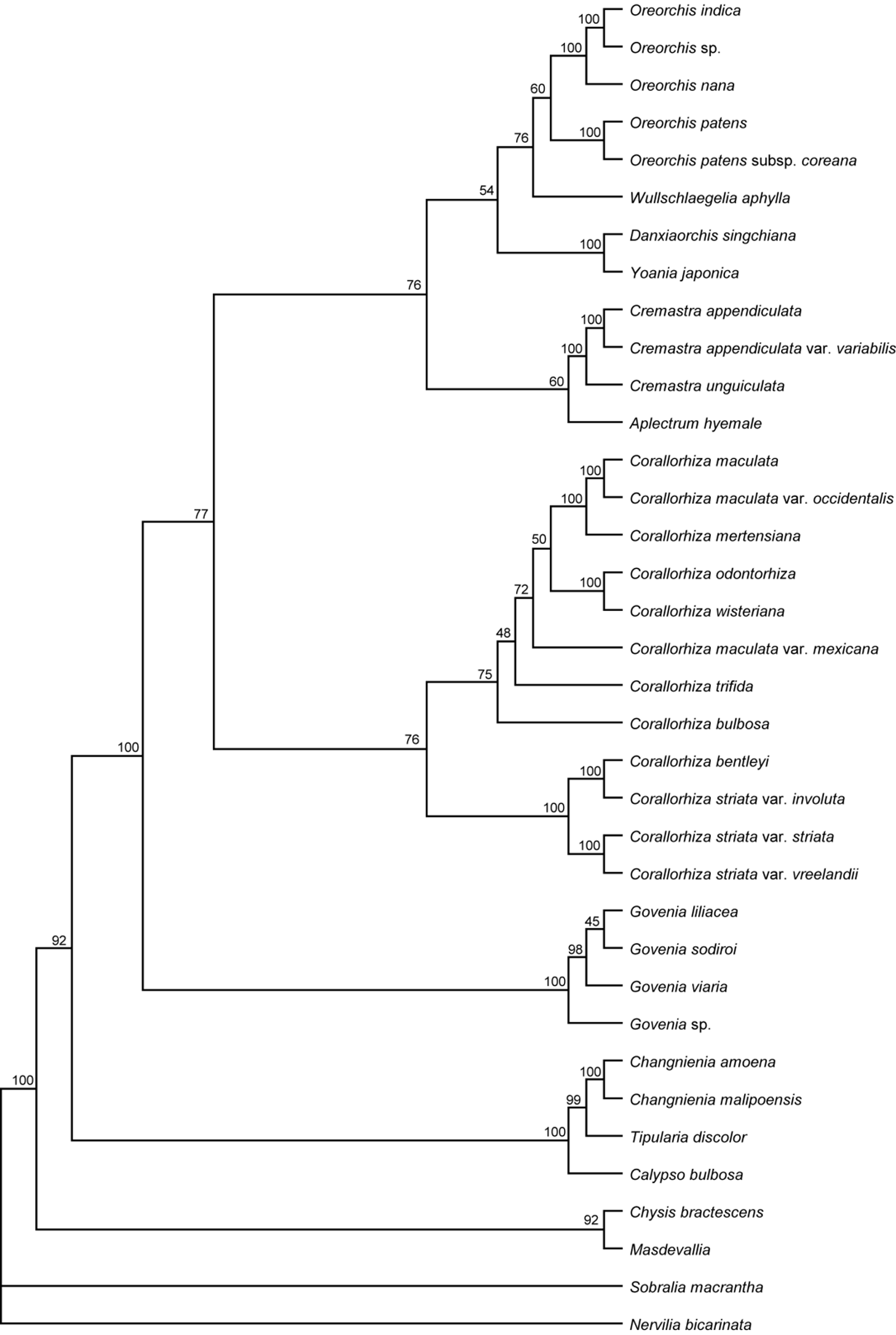

Supplement: Figure S8 — Bayesian consensus phylogram for the combined mat K and rbc L datasets, including 32 taxa of Calypsoeae. Bayesian PP (×100) is indicated above the branches. (TIF) [file pone.0060371.s008.tif]

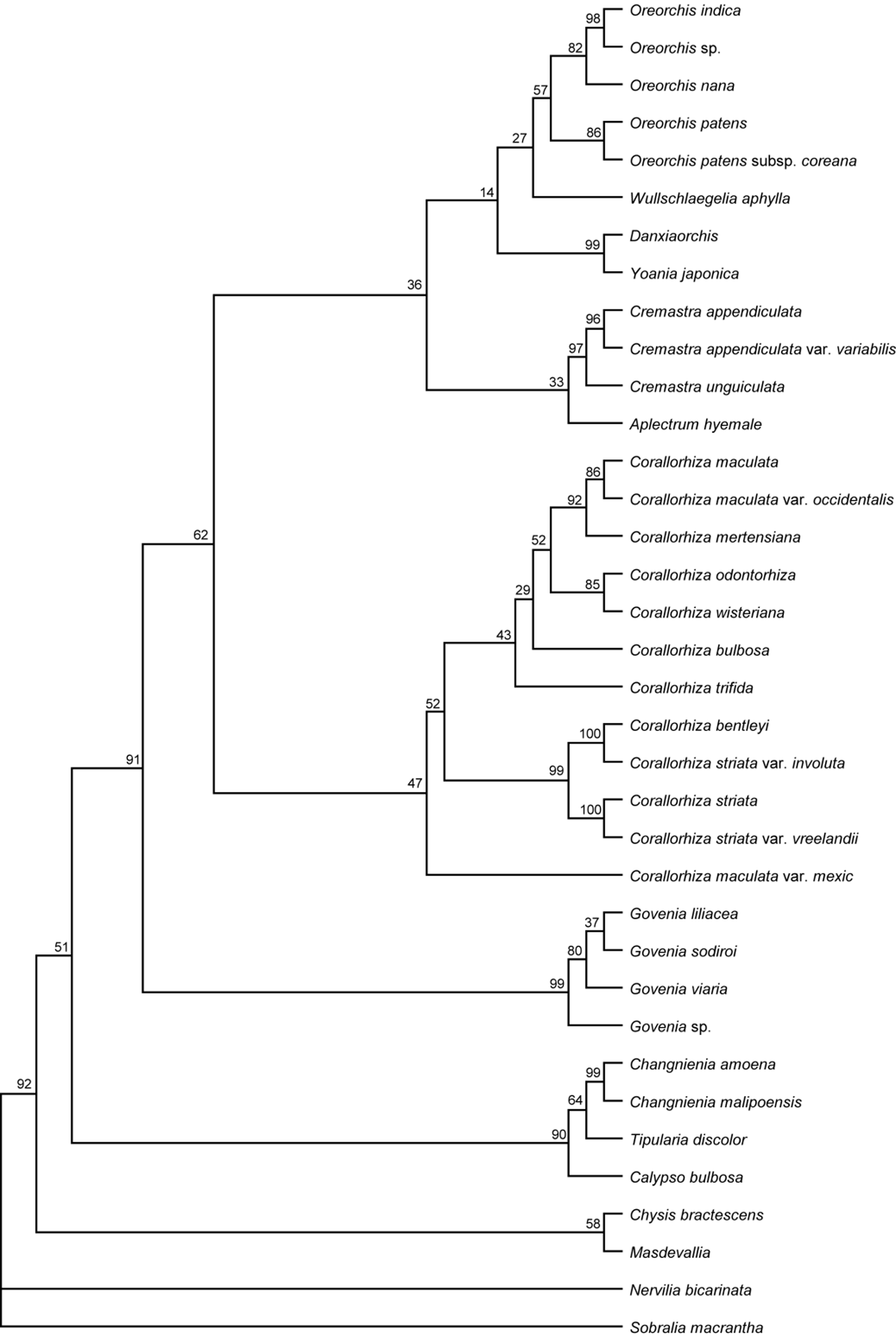

Supplement: Figure S9 — Strict consensus phylogram of most parsimonious phylograms based on the combined mat K and rbc L datasets, including 32 taxa of Calypsoeae. The bootstrap values of the MP analysis are indicated above the branches. Tree length = 931 steps; CI = 0.8217; RI = 0.8903. (TIF) [file pone.0060371.s009.tif]

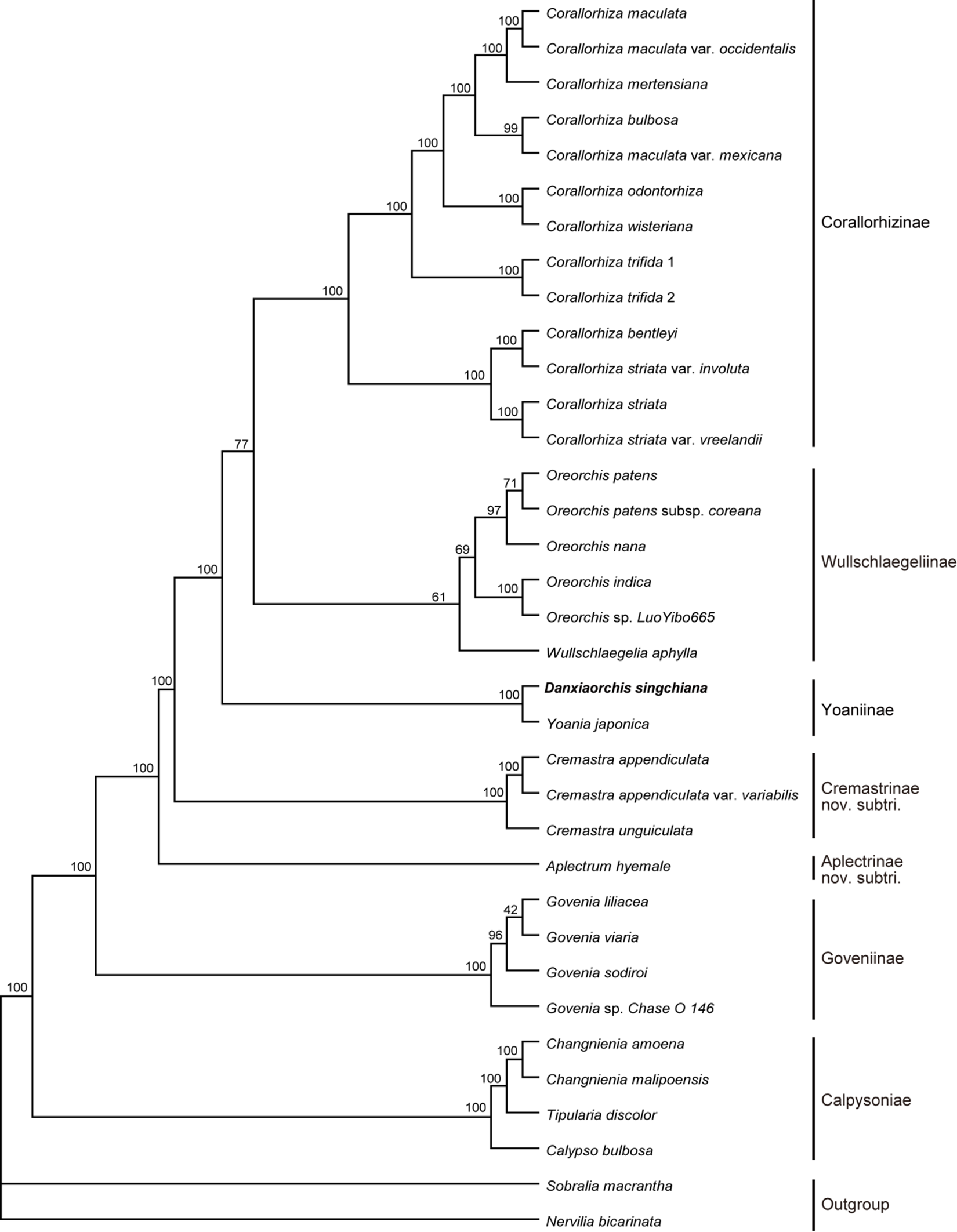

Supplement: Figure S10 — Bayesian consensus phylogram for the combined ITS, mat K, and rbc L datasets, including 33 taxa of Calypsoeae. Bayesian PP (×100) is indicated above the branches. (TIF) [file pone.0060371.s010.tif]

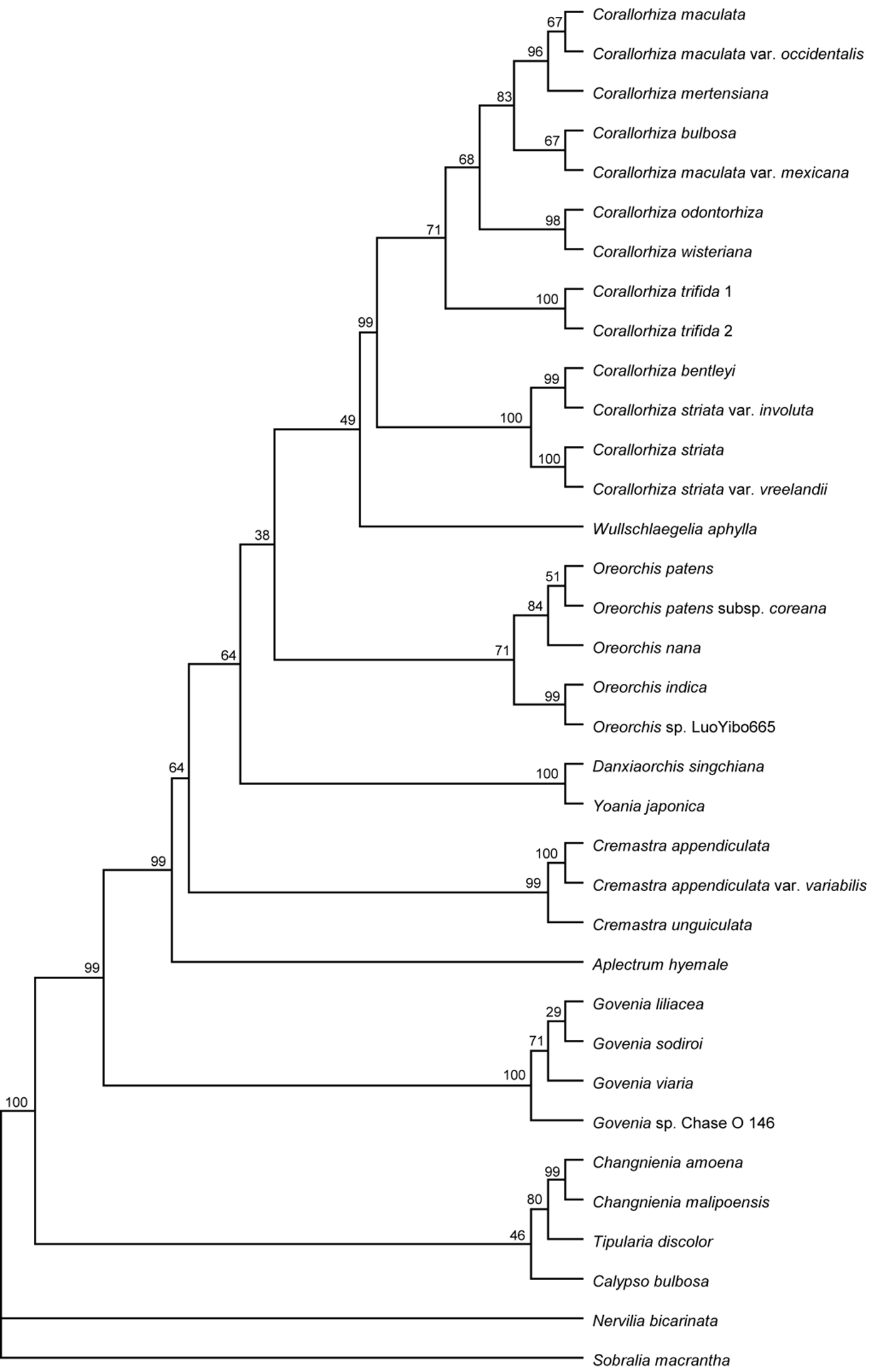

Supplement: Figure S11 — Strict consensus phylogram of most parsimonious phylograms based on the ITS, mat K, and rbc L datasets, including 33 taxa of Calypsoeae. The bootstrap values of the MP analysis are indicated above the branches. Tree length = 1505 steps; CI = 0.7980; RI = 0.8550. (TIF) [file pone.0060371.s011.tif]
