# Supplementary material for: A New Orchid Genus, Danxiaorchis, and Phylogenetic Analysis of the Tribe Calypsoeae
Source: PLoS One. 2013 Apr 4;8(4):e60371. doi: 10.1371/journal.pone.0060371 (PMC3617198; doi:10.1371/journal.pone.0060371)
Supplement: Table S1 — Samples used in Orchidaceae gene sequencing and their information. (DOC) [file pone.0060371.s012.doc]

**Table S1.** Samples used in Orchidaceae gene sequencing and their information

| **Species** | **Voucher** | **Location** | **ITS Accession number** | ***mat*K Accession number** | ***rbc*L Accession number** |
| --- | --- | --- | --- | --- | --- |
| *Altensteinia fimbriata* |  |  | AM419765.1 | EF065583.1 | FJ571315.1 |
| *Aplectrum hyemale* |  |  | EU266404.1 | EU266416.1 | FJ445516.1 |
| *Apostasia wallichii* |  |  | AY557228.1 | AY557212.1 | HM640552.1 |
| *Bifrenaria tyrianthina* |  |  | AY063419.1 | DQ210752.1 | AF074112.1 |
| *Calanthe calanthoides* |  |  | AF521063.1 | AF263632.1 | AF264159.1 |
| *Calypso bulbosa* |  |  | AF521076.1 | EF525689.1 | AF264162.1 |
| *Cattleya trichopiliochila* |  |  | AY008628.1 | AF263815.1 | AF074122.1 |
| *Changnienia malipoensis* | *Z. J. Liu 5228* | Yunnan, China | JX293179 | JX293183 | JX293188 |
| *Chiloglottis trapeziformis* |  |  | AY042153.1 | AJ310003.1 | AF074124.1 |
| *Chloraea magellanica* |  |  | AJ539523.1 | AJ543948.1 | - |
| *C. reticulata* |  |  | - | - | FJ571318.1 |
| *Cleistes rosea* |  |  | - | AJ310006.1 | AF074128.1 |
| *C. exilis* |  |  | EU498144.1 | - | - |
| *Codonorchis lessonii* |  |  | AF348005.1 | DQ414993.1 | AY381113.1 |
| *Corallorhiza wisteriana* |  |  | - | EF525703.1 | - |
| *Cranichis apiculata* |  |  | AM419784.1 | AM900819.1 | AM778148.1 |
| *C. fertilis* |  |  | AJ000137.1 | AJ310013.1 | AF074137.1 |
| *Cremastra appendiculata* var. *variabilis* |  |  | EU266414.1 | EU266421.1 | EU266439.1 |
| *Curculigo glabrescens* |  |  | - | JX294581 | JX290062 |
| *Cymbidium ensifolium* |  |  | AF284716.1 | AF263648.1 | AF074141.1 |
| *C. calceolus* |  |  | AY557232.1 | - | EF370100.1 |
| *C. passerinum* |  |  | - | AF263649.1 | - |
| *Danxiaorchis singchiana* | *Z. J. Liu 6038* | Guangdong, China | JX293178 | JX293186 | JX293187 |
| *Disa glandulosa* |  |  | DQ414873.1 | DQ415016.1 | AF274006.1 |
| *Disperis villosa* |  |  | - | EU301535.1 | - |
| *D. lindleyana* |  |  | AJ000129.1 | - | AY370651.1 |
| *Diuris sulphurea* |  |  | AY851078.1 | AJ310024.1 | AF074152.1 |
| *Dossinia marmorata* |  |  | AJ539521.1 | AJ543947.1 | AJ542405.1 |
| *Epipactis helleborine* |  |  | EF153104.1 | AF263659.1 | Z73707.1 |
| *Eria ferruginea* |  |  | AF521071.1 | EF079354.1 | AF074164.1 |
| *Eriochilus tenuis* |  |  | AF348031.1 | - | - |
| *E. cucullatus* |  |  | - | AJ310028.1 | AF074166.1 |
| *Eulophia spectabilis* |  |  | JN114508.1 | JN005458.1 | JN004436.1 |
| *Galeandra beyrichii* |  |  | EU877151.1 | - | - |
| *G. devoniana* |  |  | - | AY368408.1 | AF074171.1 |
| *Gomphichis bogotensis* |  |  | AJ539513.1 | AJ543941.1 | AJ542412.1 |
| *Gongora portentosa* |  |  | AF239389.1 | AF239485.1 |  |
| *G. amparoana* |  |  | - | - | AY368358.1 |
| *G. macrantha* |  |  | GQ328779.1 | - | - |
| *G. repens* |  |  | - | - | FJ571330.1 |
| *Goodyera pubescens* |  |  | - | AJ543954.1 | - |
| *Habenaria dives* |  |  | DQ522075.1 | - | - |
| *H. repens* |  |  | - | AJ310036.1 | AF074177.1 |
| *Holcoglossum quasipinifolium* |  |  | HQ452909 | HQ452939 | HQ452924 |
| *Hypoxis hemerocallidea* |  |  | - | - | HM640539.1 |
| *H. leptocarpa* |  |  | - | AY368375.1 | - |
| *Listera smallii* |  |  | AF521058.1 | AF263668.1 | AF074184.1 |
| *Ludisia discolor* |  |  | AJ539483.1 | AJ543911.1 | AJ542395.1 |
| *Lycaste cruenta* |  |  | AF239342.1 | AF239438.1 | AF074185.1 |
| *Masdevallia bicornis* |  |  | DQ923764.1 | - | - |
| *M. bicolor* |  |  | - | AF265447.1 | - |
| *M. infracta* |  |  | - | - | AF074189.1 |
| *Maxillaria cucullata* |  |  | DQ209997.1 | DQ210898.1 | AF074190.1 |
| *Megastylis glandulosa* |  |  | AJ539525.1 | AJ543950.1 | AJ542401.1 |
| *Microtis parviflora* |  |  | DQ104554.1 | AJ310045.1 | AF074194.1 |
| *Mormodes vinacea* |  |  | - | EF079252.1 | - |
| *M.* sp. |  |  | - | - | AF074196.1 |
| *Nervilia shirensis* |  |  | AF521066.1 | AY121735.1 | - |
| *N. bicarinata* |  |  | - | - | AF074199.1 |
| *Neuwiedia veratrifolia* |  |  | AY557227.1 | AY557211.1 | AF074200.1 |
| *Oncidium excavatum* |  |  | AF350843.1 | AY368423.1 | AF074201.1 |
| *Orchis quadripunctata* |  |  | - | AY368385.1 | - |
| *O. anatolica* |  |  | HQ657131.1 | - | - |
| *O. rotundifolia* |  |  | - | - | AY149368.1 |
| *Oreorchis indica* | *Z. J. Liu 6163* | Sichuan, China | JX293180 | JX293185 | JX293191 |
| *Pachyplectron arifolium* |  |  | AF348049.1 | AJ310051.1 | FJ571336.1 |
| *Palmorchis trilobulata* |  |  | - | AJ310052.1 | AF074206.1 |
| *Paraholcoglossum amesianum* |  |  | EU558908 | EU558882 | EU558950 |
| *Phalaenopsis equestris* |  |  | AY912225.1 | AY121744.1 | AF074211.1 |
| *Phragmipedium ecuadorense* |  |  | AY918824.1 | AY918832.1 | AY918856.1 |
| *Pleione formosana* |  |  | AF461484.1 | AF302705.1 | AF264173.1 |
| *Platanthera chlorantha* |  |  | AY704975.1 | EF612531.1 | - |
| *P. ciliaris* |  |  | - | - | AF074215.1 |
| *Platythelys querceticola* |  |  | FJ473336.1 | AY368386.1 | FJ571338.1 |
| *Pogonia ophioglossoides* |  |  | EU498161.1 | AJ310055.1 | EU498136.1 |
| *Ponthieva trilobata* |  |  | AM901013.1 | AM901011.1 | AM901012.1 |
| *Pterostylis nutans* |  |  | GQ866388.1 | GQ405616.1 | AF074224.1 |
| *Sarcoglottis acaulis* |  |  | AJ539500.1 | AJ310068.1 | AJ542424.1 |
| *Selenipedium aequinoctiale* |  |  | - | EF079360.1 | - |
| *S. chica* |  |  | - | - | AF074227.1 |
| *Sobralia macrantha* |  |  | HM854651.1 | AF263681.1 | AF074228.1 |
| *Spiranthes cernua* |  |  | EU384841.1 | AJ543917.1 | AJ542435.1 |
| *Stanhopea ecornuta* |  |  | AF239349.1 | AF239445.1 | AF074230.1 |
| *Tipularia discolor* |  |  | - | AF263685.1 | AF074234.1 |
| *Tsiorchis kimballiana* |  |  | JN106331 | JN106338 | JN106345 |
| *Vanilla planifolia* |  |  | GQ867245.1 | AJ310079.1 | FN545561.1 |
| *Wullschlaegelia aphylla* |  |  |  | AY368434.1 | AY368436.1 |
| *Yoania japonica* | *Z. J. Liu 6241* | Fujian, China | - | - | JX293190 |
| *Zygopetalum maxillare* |  |  | AY870095.1 | EU123676.1 (TRNK) | - |
| *Z. intermedium* |  |  | - | - | AF074246.1 |
