# Supplementary material for: A New Orchid Genus, Danxiaorchis, and Phylogenetic Analysis of the Tribe Calypsoeae
Source: PLoS One. 2013 Apr 4;8(4):e60371. doi: 10.1371/journal.pone.0060371 (PMC3617198; doi:10.1371/journal.pone.0060371)
Supplement: Table S2 — Samples used in Calypsoeae gene sequencing and their information. (DOC) [file pone.0060371.s013.doc]

**Table S2.** Samples used in Calypsoeae gene sequencing and their information

| **Species** | **Voucher** | **Location** | **ITS Accession**  **number** | ***mat*K Accession**  **number** | ***rbc*L Accession**  **number** |
| --- | --- | --- | --- | --- | --- |
| *Aplectrum hyemale* |  |  | EU266404.1 | EU266416.1 | FJ445516.1 |
| *Calypso bulbosa* |  |  | AF521076.1 | EF525689.1 | AF264162.1 |
| *Changnienia malipoensis* | *Z. J. Liu 5228* | Yunnan, Chian | JX293179 | JX293183 | JX293188 |
| *Chysis bractescens* |  |  | EF079363.1 | EF079351.1 | AF074126.1 |
| *Corallorhiza bentleyi* |  |  | JF319668.1 | EF525706.1 | JF319769.1 |
| *C. bulbosa* |  |  | EU391332.1 | EF525699.1 | EU391366.1 |
| *C.maculata* |  |  | EU391329.1 | EF525700.1 | EU391363.1 |
| *C. maculata* var. *mexicana* |  |  | EU391331.1 | - | EU391365.1 |
| *C. maculata* var. *occidentalis* |  |  | EU391330.1 | EF525697.1 | EU391364.1 |
| *C. mertensiana* |  |  | EU391333.1 | EF525704.1 | EU391367.1 |
| *C. odontorhiza* |  |  | EU391326.1 | EF525701.1 | EU391359.1 |
| *C.striata* var. *involuta* |  |  | EU391347.1 | EF525698.1 | FJ445565.1 |
| *C. striata* |  |  | EU391349.1 | EF525702.1 | FJ445537.1 |
| *C. striata* var. *vreelandii* |  |  | EU391352.1 | EF525705.1 | GU223951.1 |
| *C. trifida* | *Z. J. Liu 5504* | Sichuan, China | JX293181 | - | - |
| *C. trifida* |  |  | EU391324.1 | EF525695.1 | EU391357.1 |
| *C. wisteriana* |  |  | EU391327.1 | EF525703.1 | EU391361.1 |
| *Cremastra appendiculata* | *Z. J. Liu 3971* | Yunnan, China | - | JX293182 | JX293189 |
| *C. appendiculata* var. *variabilis* |  |  | EU266414.1 | EU266421.1 | EU266439.1 |
| *C. unguiculata* |  |  | EU266415.1 | EF525692.1 | - |
| *Danxiaorchis singchiana* | *Z. J. Liu 6038* | Guangdong, China | JX293178 | JX293186 | JX293187 |
| *Govenia liliacea* |  |  | AF521056.1 | AY121723.1 | - |
| *G. sodiroi* |  |  | - | EU490695.1 | - |
| *G.* sp. *Chase O-146* |  |  | EF525672.1 | EF525690.1 | AF074175.1 |
| *G. viaria* |  |  | - | EU214357.1 | - |
| *Oreorchis indica* |  |  | JX293180 | JX293184 | JX293191 |
| *Oreorchis nana* | *Z. J. Liu 5509* | Sichuan, China | - | JX293185 | JX293192 |
| *O. patens* subsp. *coreana* |  |  | EU266410.1 | EU266417.1 | - |
| *O.* sp. *LuoYibo665* |  |  | EU266413.1 | EU266420.1 | - |
| *O.* sp*. SH-2010* |  |  | - | - | AB586472.1 |
| *Tipularia discolor* |  |  | - | AF263685.1 | AF074234.1 |
| *Wullschlaegelia aphylla* |  |  | - | AY368434.1 | AY368436.1 |
| *Yoania japonica* | *Z. J. Liu 6241* | Fujian, China | - | - | JX293190 |
