# Supplementary material for: A New Orchid Genus, Danxiaorchis, and Phylogenetic Analysis of the Tribe Calypsoeae
Source: PLoS One. 2013 Apr 4;8(4):e60371. doi: 10.1371/journal.pone.0060371 (PMC3617198; doi:10.1371/journal.pone.0060371)
Supplement: Table S3 — Primers used in this study. (DOC) [file pone.0060371.s014.doc]

**Table S3.** Primers used in this study.

| **Primer** | **Sequence（5’→3’）** | **Origin** |
| --- | --- | --- |
| ITS A | GGAAGGAGAAGTCGTAACAAGG | Mike T, et al. [24] |
| ITS B | CTTTTCCTCCGCTTATTGATATG | Mike T, et al. [24] |
| *rbc*L-1F | ATGTCACCACAAACAGAAAC | Sulaiman SF, et al. [26] |
| *rbc*L-516F | TGTACTATTAAACCAAAATTGGG | This study |
| *rbc*L-880F | CAGGCTGGTACAGTAGTGGG | This study |
| *rbc*L-1285F | GCATGTGTACAAGCTCGTAATGAG | This study |
| *rbc*L-1360R | CTTCACAAGCAGCAGCTAGTTC | Reeves G., et al. [25] |
| *rbc*L-1368R | CTTTCCAAATTTCACAAGCAGCA | Reeves G., et al. [25] |
| *trn*K-2R | AACTAGTCGGATGGAGTAG | Mike T, et al. [24] |
| *mat*K-19F | CGTTCTCATATTGCACTATG | Mike T, et al. [24] |
| *mat*K-1867R | TTGCAGTTTTCATTGCACACG | Liu ZJ, et al. [30]^§^ |
| *mat*K-147F | AACAAAACTTCCTATATCCGCT | Liu ZJ, et al. [30]^§^ |
| *mat*K-1167R | CATTTGATTTCTTACTACC | Liu ZJ, et al. [30]^§^ |
| *mat*K-1149F | GGTAGTAAGAAATCAAATG | Liu ZJ, et al. [30]^§^ |
| *mat*K-969R | CTTTTCCTTGATATCGAACAT | Liu ZJ, et al. [30]^§^ |
| *mat*K-731F | AAGAAAAGATTCTTTTGGTTCC | Liu ZJ, et al. [30]^§^ |

§Additional reference

30. Liu ZJ, Chen LJ, Chen SC, Cai J, Tsai WC, et al.(2011) *Paraholcoglossum* and *Tsiorchis,* two new orchid genera established by molecular and morphological analyses of the *Holcoglossum* alliance. PLoS ONE 6 (10): e24864. DOI: 10.1371/joural.pone.0024864.
