# Supplementary material for: A New Orchid Genus, Danxiaorchis, and Phylogenetic Analysis of the Tribe Calypsoeae
Source: PLoS One. 2013 Apr 4;8(4):e60371. doi: 10.1371/journal.pone.0060371 (PMC3617198; doi:10.1371/journal.pone.0060371)
Supplement: Table S4 — Morphological data matrix for the phylogenetic analysis. (DOC) [file pone.0060371.s015.doc]

**Table S4.** Morphological data matrix for the phylogenetic analysis.

| **taxa** | **characters** |
| --- | --- |
|  | 1111111111222222222233333333334444444444555555555 |
|  | 01234567890123456789012345678901234567890123456789012345678 |
| *Alisma* | ???????????????????????????????????????????????????????0000 |
| *Altensteinia* | 01001100?0000?1000010001001000100010200000010?2001010010000 |
| *Apostasia* | 00???000?000000000000010010000000000010000000?0000000010000 |
| *Bifrenaria* | 01???0011?111????1??000000121??000????010000??2?1??????0000 |
| *Calanthe* | 01000001011012000111000110111010001120010101122102100010000 |
| *Calypso* | 10??0001011010010021000000110??00101??0100001?1?122??020000 |
| *Cattleya* | 01202001012010000011000100111010001120010001121102000110000 |
| *Changnienia* | 10??0001011010010021000000110??01001??0100001?1?122??020000 |
| *Chiloglottis* | 1?????00110?0????021000100101???00?1??100000??1???????10000 |
| *Chloraea* | 00???000?0000?1010010001001000000010200000010?1002010010000 |
| *Cleistes* | 0?????001?0?0????021000000101??001????000000??1???????10000 |
| *Codonorchis* | 11????001?0?0????0??00??00101???00?0???01000??0????????0000 |
| *Corallorhiza* | 00??0000223220222021000000110??01001??0100001?1?1?????20000 |
| *Cranichis* | 0????000100?0????02100??00101??100????0000001?1???????10000 |
| *Cremastra* | 01??0001011010010121000000110??01001??0100001?1?122??020001 |
| *Curculigo* | 0?????00201102???110000000000??000????0000?10?0?0?????20000 |
| *Cymbidium* | 01201001012010000111000100121010011?20011011122102201100000 |
| *Cypripedium* | 00???000?110000110010101010000100001000000010?0002000000000 |
| *Danxiaorchis* | 11??0000223220222021000000110??01001??1000001?1002000111110 |
| *Disa* | 10???000?0000?1010010001001002010010201000010?2001000110000 |
| *Disperis* | 1????000??000?1010?1000100100001001020100001??2001100110000 |
| *Diuris* | 10???000??000?1010010001001000000010200000010?2001010110000 |
| *Dossinia* | 0????000100?0????02100??00101??100????1010001?0????????0000 |
| *Epipactis* | 00???000?11002011001000100100000001010000001011002000010000 |
| *Eria* | 01000001012011000111000100111010001120010201121102100110000 |
| *Eriochilus* | 11????00??0?0????021000?001?1???????????0000??1???????10000 |
| *Eulophia* | 10??0001011010010021000000110??01001??0110001?1?122??020000 |
| *Galeandra* | 01???00101011????0??000100121??000???00100001?2?1??????0000 |
| *Galeola* | 00??0000223220222020000000110??00001??0010001?0?1?0???20000 |
| *Gomphichis* | 0?????00?0000?10?0010001001000100010200000010?20??????00000 |
| *Gongora* | 01???00111111????1??000?0012???000????011000??2?1??????0000 |
| *Goodyera* | 00???100?0000?1000010001001001000010201000010?2002010010000 |
| *Habenaria* | 10????00100?0????0??000100101??100????1010000?1???????10000 |
| *Hypoxis* | 0?????00200102???110000000000??000000?0000?10?0?0?????20000 |
| *Listera* | 00???000?1000?1110010001001000000010100000?1011002000000000 |
| *Ludisia* | 01000100?0000?100001000100100010001020100001012002010010000 |
| *Lycaste* | 01201001111010000111000100121010011120010011122101100110000 |
| *Masdevallia* | 01???000??011????0??000100121??000???00110001?1?1?????10000 |
| *Maxillaria* | 01201001112010000111000100121010011120010011122101100110000 |
| *Megastylis* | 0????000100?0????02100??00101??000????0010?01?0????????0000 |
| *Microtis* | 1?????001?0?0????021000000101??001????000000??1???????10000 |
| *Mormodes* | 01???00101011????1??000100121??000???00100001?2?1?????10000 |
| *Nervilia* | 00???00101100?1000110001001100100010202000010?1002010110000 |
| *Neuwiedia* | 0????000?010000000000010000000000000010000000?0000000010000 |
| *Oncidium* | 01201001112010000111000100121010011120011011122101100110000 |
| *Orchis* | 10????00100?0????0??00??00101??100????1010001?1???????10000 |
| *Oreorchis* | 01??0001011010010121000000110??01001??0100001?1?122??020000 |
| *Pachyplectron* | 0????000?00?0????02100??00101???00????1010?0??1????????0000 |
| *Palmorchis* | 0????000?110000100110000001100000001?00000000???00000110000 |
| *Paphiopedilum* | 01000000?1200?000000010101000010000100000001000002000000000 |
| *Phalaenopsis* | 01211010?12011000111000100121010011120010011122111000110000 |
| *Phragmipedium* | 0????000?1210????0??010101020??000???00000?10?0?1??????0000 |
| *Platanthera* | 10???000?1000?1010010001001002010011201000010?2001?00110000 |
| *Platythelys* | 0????100?0000????0??00??0010????0?????101000????1??????0000 |
| *Pleione* | 01???0011?101????0??00000012???000????0100001?1?1??????0000 |
| *Pogonia* | 01000000?1000?1110000000001100000001100000010??0?2000110000 |
| *Ponthieva* | 01000100?0000?1000010001001000100010200000010120???00?10000 |
| *Pterostylis* | 10???000?0000?10100100010011000000?0100000010??0020100?0000 |
| *Rhodohypoxis* | ???????????????????????????????????????????????????????0000 |
| *Sarcoglottis* | 01????00100?0????0??000100101??100????1000?0??1????????0000 |
| *Selenipedium* | 0????000?0110????0??010101020??000???00000?10?0?0??????0000 |
| *Sobralia* | 0120?000?110100000110001001110100011200002011?1002000110000 |
| *Spiranthes* | 01001100?0000?100001000100100010001020000001012001100000000 |
| *Stanhopea* | 01201001111010000111000100121010011120011011122102100110000 |
| *Tipularia* | 11??0001011010010021000000110??01001??0100001?0?122??020000 |
| *Vanilla* | 01200010?100020001101000101100000001300000010?1002000?10000 |
| *Wullschlaegelia* | 01???000?????????0??000100121??000?0??1010001?2?1??????0000 |
| *Zygopetalum* | 01201001111010000111000100121010011120010011122101100100000 |
