# Supplementary material for: A New Orchid Genus, Danxiaorchis, and Phylogenetic Analysis of the Tribe Calypsoeae
Source: PLoS One. 2013 Apr 4;8(4):e60371. doi: 10.1371/journal.pone.0060371 (PMC3617198; doi:10.1371/journal.pone.0060371)
Supplement: Table S5 — Morphological data matrix for the tribe-level phylogenetic analysis. (DOC) [file pone.0060371.s016.doc]

**Table S5.** Morphological data matrix for the tribe-level phylogenetic analysis

| **taxa** | **characters** |
| --- | --- |
|  | 11111111112222222222333333333344444444445555555555666666666 |
|  | 012345678901234567890123456789012345678901234567890123456789012345678 |
| *Aplectrum hyemale* | 01??0001011010010121000000110??01001??0100001?1?122??0200031011010101 |
| *Calypso bulbosa* | 10??0001011010010021010000110??00101??0100001?1?122??0200030111020010 |
| *Changnienia amoena* | 10??0001011010010021000000110??01001??0100001?1?122??0200000011010110 |
| *Changnienia malipoensis* | 10??0001011010010021000000110??01001??0100001?1?122??0200000011011110 |
| *Corallorchiza trifida* | 00??0000223220222021000000110??01001??0100001?1?1?????200021011010000 |
| *Corallorhiza bentleyi* | 00??0000223220222021000000110??01001??0100001?1?1?????200031011100000 |
| *Corallorhiza bulbosa* | 00??0000223220222021000000110??01001??0100001?1?1?????200021011010000 |
| *Corallorhiza maculata* | 00??0000223220222021000000110??01001??0100001?1?1?????200021011010100 |
| *Corallorhiza maculata* var. *mexicana* | 00??0000223220222021000000110??01001??0100001?1?1?????200021011010100 |
| *Corallorhiza maculata* var. *occidentalis* | 00??0000223220222021000000110??01001??0100001?1?1?????200021011010100 |
| *Corallorhiza mertensiana* | 00??0000223220222021000000110??01001??0100001?1?1?????200021011010100 |
| *Corallorhiza odontorhiza* | 00??0000223220222021000000110??01001??0100001?1?1?????200021011010000 |
| *Corallorhiza involuta* | 00??0000223220222021000000110??01001??0100001?1?1?????200031011100000 |
| *Corallorhiza striata* | 00??0000223220222021000000110??01001??0100001?1?1?????200031011100000 |
| *Corallorhiza vreelandii* | 00??0000223220222021000000110??01001??0100001?1?1?????200031011100000 |
| *Corallorhiza trifida* | 00??0000223220222021000000110??01001??0100001?1?1?????200021011010000 |
| *Corallorhiza wisteriana* | 00??0000223220222021000000110??01001??0100001?1?1?????200021011010000 |
| *Cremastra appendiculata* | 01??0001011010010121000000110??01001??0100001?1?122??0200011011010000 |
| *Cremastra appendiculata* var. *variabilis* | 01??0001011010010121000000110??01001??0100001?1?122??0200011011010010 |
| *Cremastra unguiculata* | 01??0001011010010121000000110??01001??0100001?1?122??0200011011010000 |
| *Danxiaorchis singchiana* | 11??0000223220222021000000110??01001??1000001?10020001111111011110100 |
| *Govenia liliacea* | 01??0000211010010121000000110??01001??0100001?1?122??0200031110010010 |
| *Govenia sodiroi* | 01??0000211010010121000000110??01001??0100001?1?122??0200031110010010 |
| *Govenia* sp. *Chase O 146* | 01??0000211010010121000000110??01001??0100001?1?122??0200031110??0??0 |
| *Govenia viaria* | 01??0000211010010121000000110??01001??0100001?1?122??0200031110010010 |
| *Nervilia bicarinata* | 00???00101100?1000110001001100100010202000010?10020101100031?0?000100 |
| *Oreorchis indica* | 11??0001011010010121000000110??11001??0100001?1?122??0200031011010100 |
| *Oreorchis nana* | 11??0001011010010121000000110??11001??0100001?1?122??0200031011010100 |
| *Oreorchis patens* | 11??0001011010010121000000110??11001??0100001?1?122??0200031011010100 |
| *Oreorchis patens* subsp. *coreana* | 11??0001011010010121000000110??11001??0100001?1?122??0200031011010100 |
| *Oreorchis* sp. *Luo Yibo 665* | 11??0001011010010121000000110??11001??0100001?1?122??0200031011010?00 |
| *Cremastra unguiculata* | 01??0001011010010121000000110??01001??0100001?1?122??0200011011011000 |
| *Sobralia macrantha* | 0120?000?110100000110001001110100011200002011?10020001100031?1?010000 |
| *Tipularia discolor* | 11??0001011010010021000000110??01001??0100001?0?122??0200010111101000 |
| *Wullschlaegelia aphylla* | 01???000?????????0??000100121??000?0??1010001?2?1??????00031001010000 |
| *Dactylostalix* | 01??0000211010010021000000110??00001??0100001?0?122??0200030011000110 |
| *Ephippianthus* | 10??0000111010010021000000110??00001??0100001?0?122??0200031011010000 |
| *Yoania japonica* | 10??0000233220222021000000110??00001??0000001?1?122??0200011011010000 |
