# Supplementary material for: A New Orchid Genus, Danxiaorchis, and Phylogenetic Analysis of the Tribe Calypsoeae
Source: PLoS One. 2013 Apr 4;8(4):e60371. doi: 10.1371/journal.pone.0060371 (PMC3617198; doi:10.1371/journal.pone.0060371)
Supplement: Table S6 — Statistics from the analyses of various datasets. (DOC) [file pone.0060371.s017.doc]

**Table S6.** Statistics from the analyses of various datasets.

| **Information** | **Calypsoeae phylograms** | | | **Orchidaceae phylograms** | |
| --- | --- | --- | --- | --- | --- |
|  | **ITS** | ***mat*K & *rbc*L** | **ITS, *mat*K & *rbc*L** |  | **ITS, *mat*K & *rbc*L** |
| No. of taxa | 27 | 34 | 35 | 71 | |
| Aligned length | 682 | 3009 | 3691 | 3806 | |
| No. variable characters | 321 | 686 | 1007 | 2128 | |
| No. informative characters (%) | 179 (26.25%) | 306 (10.17%) | 485  (13.14%) | 1518  (39.88%) | |
| Tree length | 555 | 931 | 1505 | 10188 | |
| Consistency index (CI) | 0.8153 | 0.8217 | 0.7980 | 0.3728 | |
| Retention index (RI) | 0.8145 | 0.8903 | 0.8550 | 0.5857 | |
| Rescaled consistency index (RC) | 0.6641 | 0.7315 | 0.6823 | 0.2184 | |
