# Supplementary material for: A New Orchid Genus, Danxiaorchis, and Phylogenetic Analysis of the Tribe Calypsoeae
Source: PLoS One. 2013 Apr 4;8(4):e60371. doi: 10.1371/journal.pone.0060371 (PMC3617198; doi:10.1371/journal.pone.0060371)
Supplement: Table S7 — Best-fit model and parameter for each Orchidaceae dataset. (DOC) [file pone.0060371.s018.doc]

**Table S7**. Best-fit model and parameter for each Orchidaceae dataset

| **Region** | **AIC select model** | **Base frequencies** | | | | | **substitution model(rate matrix)** | | | | | | | | **I** | | **G** | |
| --- | --- | --- | --- | --- | --- | --- | --- | --- | --- | --- | --- | --- | --- | --- | --- | --- | --- | --- |
|  |  | **A** | **C** | **G** | **T** |  | | **A-C** | **A-G** | **A-T** | **C-G** | **C-T** | **G-T** |  | |  | |  |
| ITS | TrN+I+G | 0.2307 | 0.2377 | 0.2592 | 0.2724 | | 1.0000 | | 3.3453 | 1.0000 | 1.0000 | 3.9282 | 1.0000 | | 0.1093 | | 1.3333 | |
| *mat*K | TVM+I+G | 0.3506 | 0.1256 | 0.1313 | 0.3926 | | 1.3911 | | 1.7068 | 0.1754 | 0.4969 | 1.7068 | 1.0000 | | 0.1071 | | 1.2613 | |
| *rbc*L | GTR+I+G | 0.2868 | 0.1826 | 0.2360 | 0.2946 | | 1.4600 | | 3.0809 | 0.5896 | 0.8706 | 4.8115 | 1.0000 | | 0.4756 | | 0.7061 | |
| Combined | GTR+I+G | 0.2579 | 0.2111 | 0.2378 | 0.2933 | | 1.3345 | | 2.3857 | 0.6980 | 0.4229 | 2.6901 | 1.0000 | | 0.2120 | | 0.4854 | |
