# Supplementary material for: A New Orchid Genus, Danxiaorchis, and Phylogenetic Analysis of the Tribe Calypsoeae
Source: PLoS One. 2013 Apr 4;8(4):e60371. doi: 10.1371/journal.pone.0060371 (PMC3617198; doi:10.1371/journal.pone.0060371)
Supplement: Table S8 — Best-fit model and parameter for each Calypsoeae dataset. (DOC) [file pone.0060371.s019.doc]

**Table S8**. Best-fit model and parameter for each Calypsoeae dataset

| **Region** | **AIC select model** | **Base frequencies** | | | | | **substitution model(rate matrix)** | | | | | | | | **I** | | **G** | |
| --- | --- | --- | --- | --- | --- | --- | --- | --- | --- | --- | --- | --- | --- | --- | --- | --- | --- | --- |
|  |  | **A** | **C** | **G** | **T** |  | | **A-C** | **A-G** | **A-T** | **C-G** | **C-T** | **G-T** |  | |  | |  |
| ITS | GTR+G | 0.2245 | 0.2614 | 0.3061 | 0.2081 | | 0.7068 | | 2.3417 | 1.9094 | 0.2817 | 4.0349 | 1.0000 | | 0.0000 | | 0.7426 | |
| *mat*K | K81uf+G | 0.3132 | 0.1659 | 0.1479 | 0.3730 | | 1.0000 | | 1.4065 | 0.1861 | 0.1861 | 1.4065 | 1.0000 | | 0.0000 | | 0.7336 | |
| *rbc*L | TIM+G | 0.2841 | 0.1847 | 0.2327 | 0.2985 | | 1.0000 | | 3.6094 | 0.5557 | 0.5557 | 4.9094 | 1.0000 | | 0.0000 | | 0.3351 | |
| Combined | TVM+I+G | 0.2757 | 0.1928 | 0.2082 | 0.3233 | | 1.4650 | | 2.8308 | 0.6210 | 0.4636 | 2.8308 | 1.0000 | | 0.3102 | | 0.7983 | |
