# Supplementary material for: A New Orchid Genus, Danxiaorchis, and Phylogenetic Analysis of the Tribe Calypsoeae
Source: PLoS One. 2013 Apr 4;8(4):e60371. doi: 10.1371/journal.pone.0060371 (PMC3617198; doi:10.1371/journal.pone.0060371)
Supplement: Morphological Character Codes S1 — (DOC) [file pone.0060371.s020.doc]

**Morphological character codes S1**

0. root tubers 0=absent, 1=present

1. root epidermis 0=rhizodermis, 1=velamen

2. exodermis 0=unthickened, 1=uniformly thickened, 2=outer walls thickened

3. exodermal cell shape 0=±isodiametric, 1=elongate

4. velamen cell thickenings 0=absent, 1=linear, 2=circular

5. spiranthosomes 0=absent, 1=present

6. growth pattern 0=sympodial, 1=monopodial

7. thickened stem 0=absent, 1=present

8. number of thickened internodes 0=several, 1=one

9. phyllotaxy 0=spiral, 1=distichous

10. leaf morphology 0=flat non-plicate, 1=plicate, 2=conduplicate

11. winter leaf 0=absent, 1=present

12. leaf articulation 0=absent, 1=present

13. stigmata 0=conical, 1=spherical, 2=absent

14. leaf fiber bundles 0=present, 1=absent

15. leaf abaxial epidermal cells 0=straight, 1=wavy

16. subsidiary cells 0=present, 1=not distinguishable

17. inflorescence position 0=terminal, 1=lateral

18. floral abscission 0=absent, 1=present, ovary not stalked, 2=present, ovary stalked

19. perianth abscission 0=present, 1=absent

20. calyculus 0=absent, 1=vanilloid, 2=polystachyoid

21. slipper-shaped labellum 0=absent, 1=present

22. apiculate perianth 0=absent, 1=present

23. carinate petals 0=present, 1=absent

24. lip-column marginal adnation 0=absent, 1=present

25. dorsal median stamen 0=present, 1=absent

26. lateral inner stamens 0=present, 1=absent

27. anther orientation 0=erect, 1=bending late, 2=bending early

28. operculate anther 0=absent, 1=present

29. Endothecial thickenings 1 0=other, 1=intermediate, 2=type II

30. Endothecial thickenings 2 0=other, 1=type III/IV

31. basal caudicles 0=absent, 1=present

32. hammer stipe 0=absent, 1=present

33. tegula 0=absent, 1=present

34. pollen unit 0=monad, 1=tetrad

35. pollen tectum 0=reticulate, 1=smooth

36. pollen apertures 0=colpate/sulcate, 1=porate, 2=inaperturate, 3=polyporate

37. operculate colpus 0=absent, 1=present

38. massulae 0=absent, 1=orchidoid, 2=epidendroid, 3=arethusoid

39. pollinium texture 0=granular, 1=solid

40. pollinium number= 2 0=absent, 1=present

41. pollinium number= 8 0=absent, 1=longitudinal, 2=transverse

42. pollinium orientation 0=juxtaposed, 1=superposed

43. ovary locule number 0=one, 1=three

44. stigma 0=protruded, 1=sunken

45. stigma receptive cells 0=various other, 1=finger, 2=prosenchymatic

46. viscidium 0=none, 1=diffuse, 2=detachable

47. endocarpic trichomes 0=absent, 1=present

48. seed laterally compressed walls 0=absent, 1=present

49. seed testa cell shape 0=all isodiametric, 1=end isodiametric, middle elongate, 2=all elongate

50. seed striations 0=absent, 1=transverse/reticulate, 2=longitudinal

51. seed intercellular spaces 0=absent, 1=present

52. seed wax caps 0=absent, 1=present

53. seed covered cell border 0=absent, 1=present

54. pollinium shape (dorsal profile) 0=rhomboid, 1=clavate-obovoid

55. dust seed 0=present, 1=absent

56. lip with two sacs 0=present, 1=absent

57. seed with ensosperm 0=present, 1=absent

58. flower tubular 0=absent, 1=present

**The following characters used in the tribe-level analyses only.**

59. Labellum attachment 0=spur, 1=sac, 2=mentum, 3=absent

60. flower number 0=single, 1=more than 1 flower

61. pollinium number 0=2, 1=4,2=8

62. root velamen type 0=Cybidium type, 1=Calanthe type

63. labellum thickened 0=absent, 1=present

64. labellum shape 0=boat shaped or concave, 1=flattend, 2=slipper

65. spot on leaf 0=absent, 1=present

66. lip 3-lobed 0=absent, 1=present

67. column wing 0=absent, 1=present

68. White veins on leaf 0=absent, 1=present
